# Genome-wide association study identifies novel loci associated with skin autofluorescence in individuals without diabetes

**DOI:** 10.1186/s12864-022-09062-x

**Published:** 2022-12-19

**Authors:** Charlotte E. Vollenbrock, Delnaz Roshandel, Melanie M. van der Klauw, Bruce H. R. Wolffenbuttel, Andrew D. Paterson

**Affiliations:** 1 Department of Endocrinology, University of Groningen, University Medical Center Groningen, Groningen, The Netherlands; 2 Program in Genetics and Genome Biology, Hospital for Sick Children, Toronto, ON Canada; 3 Divisions of Biostatistics and Epidemiology, Dalla Lana School of Public Health, University of Toronto, Toronto, ON Canada

**Keywords:** Skin Autofluorescence, Genome-wide association study, Screening, Single nucleotide polymorphism, Coffee, Skin Pigmentation

## Abstract

**Background:**

Skin autofluorescence (SAF) is a non-invasive measure reflecting accumulation of advanced glycation endproducts (AGEs) in the skin. Higher SAF levels are associated with an increased risk of developing type 2 diabetes and cardiovascular disease. An earlier genome-wide association study (GWAS) revealed a strong association between *NAT2* variants and SAF. The aim of this study was to calculate SAF heritability and to identify additional genetic variants associated with SAF through genome-wide association studies (GWAS).

**Results:**

In 27,534 participants without diabetes the heritability estimate of lnSAF was 33% ± 2.0% (SE) in a model adjusted for covariates. In meta-GWAS for lnSAF five SNPs, on chromosomes 8, 11, 15 and 16 were associated with lnSAF (P < 5 × 10^–8^): 1. rs2846707 (Chr11:102,576,358,C > T), which results in a Met30Val missense variant in *MMP27* exon 1 (NM_022122.3); 2. rs2470893 (Chr15:75,019,449,C > T), in intergenic region between *CYP1A1* and *CYP1A2*; with attenuation of the SNP-effect when coffee consumption was included as a covariate; 3. rs12931267 (Chr16:89,818,732,C > G) in intron 30 of *FANCA* and near *MC1R*; and following conditional analysis 4. rs3764257 (Chr16:89,800,887,C > G) an intronic variant in *ZNF276*, 17.8 kb upstream from rs12931267; finally, 30 kb downstream from *NAT2* 5. rs576201050 (Chr8:18,288,053,G > A).

**Conclusions:**

This large meta-GWAS revealed five SNPs at four loci associated with SAF in the non-diabetes population. Further unravelling of the genetic architecture of SAF will help in improving its utility as a tool for screening and early detection of diseases and disease complications.

**Supplementary Information:**

The online version contains supplementary material available at 10.1186/s12864-022-09062-x.

## Background

Advanced glycation endproducts (AGEs) are irreversibly glycated proteins that result from complex reactions between sugars and proteins [1]. AGEs are formed and accumulate in the body during life and accumulation is amplified under conditions of glycaemic stress [2].

The accumulation of AGEs can be evaluated non-invasively by measuring skin autofluorescence (SAF) with the AGE Reader [3, 4]. The method is based on the fact that AGEs present in dermal tissue exhibit fluorescence characteristics, they absorb the illuminated light at a particular wavelength and subsequently emit light of longer wavelength. Previous studies have shown predictive value of SAF in individuals both with- and without diabetes. In non-diabetic individuals SAF is a predictor for the development of type 2 diabetes, cardiovascular events and mortality and in individuals with both type 1 and type 2 diabetes SAF predicts diabetes-related complications and mortality [5–11]. A recent study reported that SAF may also predict the occurrence of cancer in individuals with type 2 diabetes, thereby suggesting the possibility that SAF may be used in selection of type 2 diabetes individuals for cancer screening [12].

In addition to SAF, measurements performed with the AGE Reader (Diagnoptics Technologies, Groningen, the Netherlands) yield a value for skin reflectance (SR). SR is a proxy for skin colour and pigmentary phenotype, light skin reflects more light and therefore individuals with light skin have higher SR values. Pigmentary characteristics are associated with susceptibility for melanoma [13].

Variation in SAF is dependent on environmental influences such as smoking and coffee consumption and is, in part, explained by genetic variation. Heritability of serum AGEs were estimated to be 63–74% [14, 15]. Previous genome-wide association studies (GWAS) have revealed two genetic loci associated with SAF [16, 17]. The first study identified a SNP in *NAT2* which is known to tag the NAT2 acetylator phenotype, and explains around 3% of the SAF variance [16]. The second study, in individuals with type 1 diabetes, demonstrated a locus on chromosome 1 which was associated with skin intrinsic fluorescence, a parameter comparable to SAF [17]. Most of the genetic basis of SAF remains undiscovered.

The aim of the current study was to calculate SAF heritability and to identify additional genetic loci associated with SAF by performing GWAS in non-diabetic individuals of the population-based Lifelines Cohort Study.

## Methods

### Study design

First, we calculated genetic heritability of SAF. Subsequently, we performed a meta-GWAS of skin autofluorescence (SAF) on genotyped and imputed SNPs from all non-diabetic individuals in whom both results of measurement of SAF and genetic data were available (*n* = 27,534). Meta-analysis of the data was performed because genotyping of two subgroups from the Lifelines cohort was performed using different arrays, i.e. the Illumina CytoSNP 12v2 chip and the Illumina GSA chip. Genome wide significantly associated SNPs were reported. For the SNPs on chromosome 16, in a region known for its association with skin colour, we examined Reflectance-SNP interaction. In addition, for the SNP on chromosome 15, which showed a clear attenuation of the *P-*value when coffee consumption was added as a covariate to the model, we performed further investigation. First, we examined SNP-covariate interaction and in a post-hoc analysis we examined SNPs associated with coffee consumption in previous genetic studies for association with SAF. For all genome wide significantly associated loci single-tissue expression quantitative trait loci (eQTLs) were examined and reported [18].

All previously published loci for skin fluorescence were tested for association with SAF. We conducted a GWAS of SR and calculated shared heritability of SAF and SR. All signals identified for SR were tested for association with SAF and vice versa, all identified SNPs associated with SAF were tested for association with SR. All individuals were of European descent, following exclusion of non-European descent using principal component analyses.

### Study participants

Participants from the Lifelines Cohort Study, a large population-based study in the northern region of the Netherlands (Additional File 1: Supplementary Methods), were included [19]. All individuals provided written informed consent before participating in the study, which was approved by the Medical Ethics Review Committee of the University Medical Center Groningen. At baseline, both physical and laboratory examination were performed, and extensive questionnaire data were collected [19].

### Genotyping and imputation

The first set of participants of the Lifelines Cohort Study comprised 10,110 individuals with SAF measurements available, who were genotyped with the Illumina CytoSNP 12v2 (these overlap with 8,721 subjects previous reported by Eny et al., [16]). An additional set of 17,424 participants with available SAF measurements were genotyped using the Illumina Infinium Global Screening Array-24 (GSA) version 1 chip (Additional File 1: Supplementary Methods; Additional File 2: Figure S1).

Prior to imputation, genotyped SNPs with a call rate < 99%, monomorphic SNPs and SNPs with a Hardy–Weinberg equilibrium (HWE) *p* value < 10^−6^, were excluded. Subjects with a call rate < 99%, heterozygosity rate > 4 standard deviations (SD) from the mean, subjects with mismatch between self-reported sex and sex chromosome composition and outliers in principal component analyses were subsequently excluded (Additional File 2: Figure S1).

Both datasets were separately phased using SHAPEIT2 and imputed to the Haplotype Reference Consortium (HRC) panel, build GRCh37 [20]. All well-imputed SNPs (INFO ≥ 80%) and minor allele frequency (MAF) > 0.1% were included in the analyses.

### Phenotype and covariates measurement

At baseline examination, participants completed a self-administered questionnaire on medical history, past and current diseases, and health behaviour. Information regarding smoking behaviour (never, former and current smoking), as well as coffee consumption (cups/day), was collected by questionnaire [21]. Weight was measured to the nearest 0.1 kg and height to the nearest 0.5 cm, with participants wearing light clothing and no shoes. BMI was calculated as weight/height^2^ in kg/m^2^.

SAF was measured on the volar side of the forearm with the AGE Reader (Diagnoptics, Groningen, The Netherlands), using emitted light from an internal UVA blacklight source in the range 350–420 nm with a peak around 370 nm [22]. An internal spectrometer measured emitted and reflected light over 420–600 nm and 300–420 nm, respectively. Additionally, a reflectance spectrum was obtained using light from a white LED. SAF was calculated with AGE Reader software (v2.3) from the ratio between the emitted and the reflected light with skin colour variation taken into account [23]. Skin reflectance (SR) was the mean skin reflectance determined with the UVA excitation source in the range 350–420 nm.

Blood samples were taken in the fasting state between 08:00 and 10:00 h and transported to the laboratory facility at room temperature or 4 °C, depending on the sample requirements. On the same day, HbA1c (EDTA-anticoagulated) was analysed using an NGSP-certified turbidimetric inhibition immunoassay on a Cobas Integra 800 CTS analyser (Roche Diagnostics Nederland, Almere, The Netherlands). Serum creatinine was measured on a Roche Modular P chemistry analyser (Roche, Basel, Switzerland) and renal function was calculated as estimated glomerular filtration rate (eGFR) with the formula developed by the Chronic Kidney Disease Epidemiology Collaboration (CKD-EPI) [24].

By definition, this general population cohort did include individuals with the metabolic syndrome or existing cardiovascular disease. Exclusion criteria for the present analyses were a SAF value higher than 4.5 AU, impaired glucose tolerance defined as elevated fasting blood glucose ≥ 7.0 mmol/l and/or HbA1c ≥ 6.5% (48 mmol/mol) or a previous diabetes diagnosis, and severely impaired renal function indicated by eGFR < 30 ml/min/1.73m^2^ (Additional file 2: Figure S1).

### Statistical analysis

Heritability of SAF was tested by estimation of the SNP heritability through variance componence analysis using the BOLT- REML algorithm [25]. Using a Monte Carlo average information restricted maximum likelihood algorithm, BOLT-REML is a computationally efficient program for estimation of trait heritability and shared heritability. Distribution of SAF values was right skewed, therefore natural log transformed SAF (lnSAF) was chosen as the outcome in the analyses. We calculated heritability for lnSAF separately without covariates in the model and in a model adjusted for sex, smoking status, mode of inclusion into the Lifelines cohort, age, age^2^, BMI and eGFR (Additional File 1: Supplementary Methods). The GSA cohort was used to calculate the heritability estimates since it has highest number of subjects with SAF measurements.

The primary outcome of GWAS was lnSAF. SNPs were tested for association with SAF under an additive genetic model in a linear mixed model, using BOLT-LMM v2.3.4 [25]. In this model a genetic relationship matrix is calculated prior to analyses for which the model is adjusted to correct for relatedness and population structure within the cohort.

Since genetic variants may be associated with SAF through effects on covariates associated with SAF such as HbA1c, coffee consumption and SR, covariates were sequentially added in the GWAS models. Model 1 was adjusted for sex, smoking status, mode of inclusion into the Lifelines cohort, age, age^2^, body mass index (BMI), eGFR, and the *NAT2* rs1495741 (indicators for homozygosity for the G allele and heterozygosity) [16]. In model 2 we adjusted for all parameters of model 1 and HbA1c. In model 3, in addition to the covariates of model 2, coffee drinking status (yes/no) and daily coffee consumption in cups per day were added. In model 4 SR was included as an additional covariate.

An additional GWAS was performed for SR, which is a proxy for skin colour/pigmentation. The model was adjusted for age, sex, smoking group, source of inclusion, month of testing, BMI and eGFR. Shared heritability of SAF and SR was calculated in a model without covariates, and in a model adjusted for sex, smoking status, source of inclusion, age, age^2^, BMI and eGFR. Meta-GWAS of the CytoSNP and GSA cohort was performed using METAL v1.5 with the STDERR method which uses effect size estimates and standard errors [26]. Double GC correction was not performed. A *P*-value < 5 × 10^−8^ was considered genome-wide significant. For visualisation of the GWAS results Manhattan and QQ plots were created in R version 4.0.3 using the ‘qqman’ package [27, 28]. In the QQ plot lambda genomic control (λgc) values are visualised [29]. In addition, regional plots were generated using LocusZoom [21, 30].

SNP-covariate interactions were tested in a linear regression model in R version 4.0.3 [27].

Conditional analysis was performed for all novel loci separately added as a covariate to GWAS model 4, in order to test whether multiple loci in the same region were independently associated. When multiple loci within a genetic region were found, linkage disequilibrium (LD) and recombination rates were examined in the population genotyped on the GSA chip using PLINK v2.0-alpha2 [31]. GWAS model 5 for lnSAF was adjusted for all covariates in model 4 and all novel identified loci to increase power for detection of additional loci.

## Results

In total, 27,534 participants from the Lifelines Cohort Study, a large population-based study in the northern region of the Netherlands (Additional File 1: Supplementary Methods; Additional File 2: Figure S1) were available for analysis. Descriptive statistics of the participants are summarized in Table 1. Mean age of the participants was 44 years and 40.9% was male, with a median BMI of 25.0 [22.8, 27.6] kg/m^2^, median eGFR 99 [88, 109] ml/min/1.73m^2^ and median coffee consumption of 3.3 [1.9, 4.7] cups per day. Median SR was 0.20 [0.16, 0.25], a Hexbin plot for lnSAF and SR is depicted in Additional File 3: Figure S2.

**Table 1:** Descriptive statistics of the 27,534 participants

	**GSA**		**CytoSNP**	
	***N* = 17,424**	**M**	***N* = 10,110**	**M**
Skin autofluorescence (SAF), AU	1.79 [1.54, 2.09]	0	1.97 [1.71, 2.28]	0
lnSAF	0.59 ± 0.22	0	0.68 ± 0.21	0
Skin reflectance, %	0.20 [0.16, 0.25]	0	0.19 [0.15, 0.23]	0
Age, yrs	42 [32, 51]	0	48 [41, 55]	0
Male sex, n (%)	7092 (40.7%)	0	4173 (41.3%)	0
Smoking status		96		126
*non-smoker*	11,269 (65.0%)		6083 (60.9%)	
*previous smoker*	2835 (16.4%)		1753 (17.6%)	
*current smoker*	3224 (18.6%)		2148 (21.5%)	
BMI, kg/m^2^	25.0 [22.8, 27.6]	2	25.8 [23.5, 28.4]	3
eGFR, ml/min/1.73m^2^	99 [88, 109]	27	94 [83, 104]	26
HbA1c, %	5.4 ± 0.3	67	5.5 ± 0.3	23
HbA1c, mmol/mol	36 ± 3	67	36 ± 3	23
Coffee drinker (> 1 cup/week)	15,114 (87.4%)	127	9238 (93.5%)	235
Daily coffee consumption, cups/day	3.3 [1.9, 4.7]	127	3.7 [2.3, 5.1]	235
rs1495741 (NAT2-metabolisation status)		0		0
*GG (fast)*	921 (5.3%)		498 (4.9%)	
*GA (intermediate)*	6172 (35.4%)		3596 (35.6%)	
*AA (slow)*	10,331 (59.3%)		6016 (59.5%)	
Inclusion mode		0		0
*Family doctor*	7627 (43.8%)		6218 (61.5%)	
*Included family members*	7393 (42.4%)		3131 (31.0%)	
*Self-registered*	2404 (13.8%)		761 (7.5%)	

*M*, number of subjects with missing data

Values are counts (%), or mean ±SD for normally distributed variables, or median [25^th^, 75^th^ quartiles] for non-normally distributed variables

No participant reported an earlier diagnosis of diabetes or had elevated fasting blood glucose following exclusion of individuals with fasting glucose ≥ 7.0 mmol/l and/or HbA1c ≥ 6.5%

Heritability estimates.

The heritability estimate of lnSAF was 20 ± 2% (SE) without covariates. Adjusted for sex, smoking group, source of inclusion, age, age^2^, BMI and eGFR, the heritability estimate of lnSAF was 33 ± 2% (SE) in the GSA data. Heritability of SR without covariates in the model was 35 ± 2%. Shared heritability of SAF and SR without covariates was 33 ± 6%. Heritability of SR adjusted for sex, smoking group, source of inclusion, age, age^2^, BMI and eGFR was 37 ± 2% (SE). Shared heritability of SAF and SR in the model adjusted for the aforementioned covariates was 24 ± 4% (SE).

Genome-wide association analyses.

**Fig. 1:**
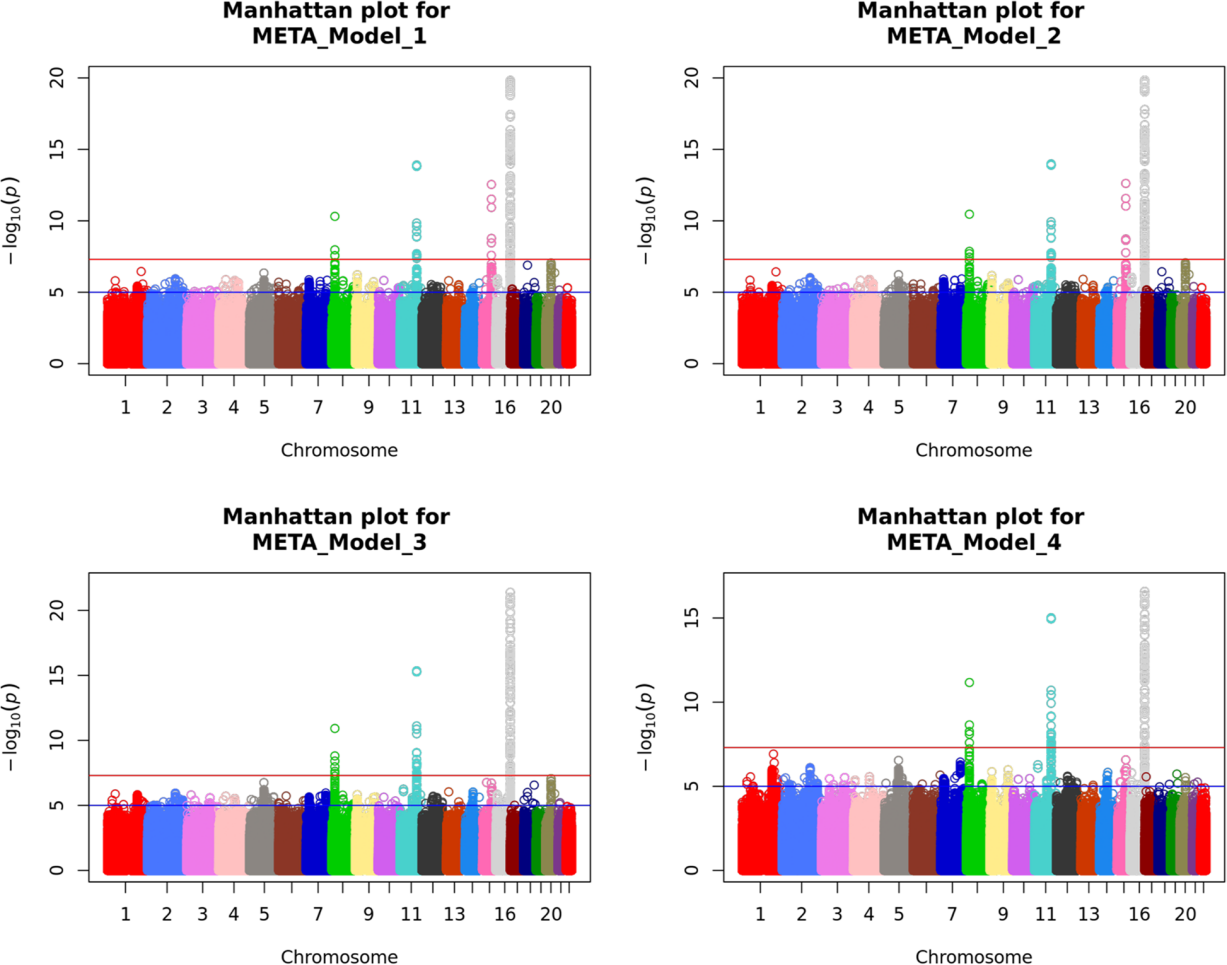
Manhattan plot of genome-wide p- values of association for Skin Autofluoresence in meta-analyses of all models Legends: On the x-axis chromosome numbers, on the y-axis the -log10 *P*-values. The horizontal red line represents the genome-wide significance threshold at *p* < 5 × 10 -8, the horizontal blue line represents the *p* < 1 × 10 -5, for suggestive associations

**Fig. 2:**
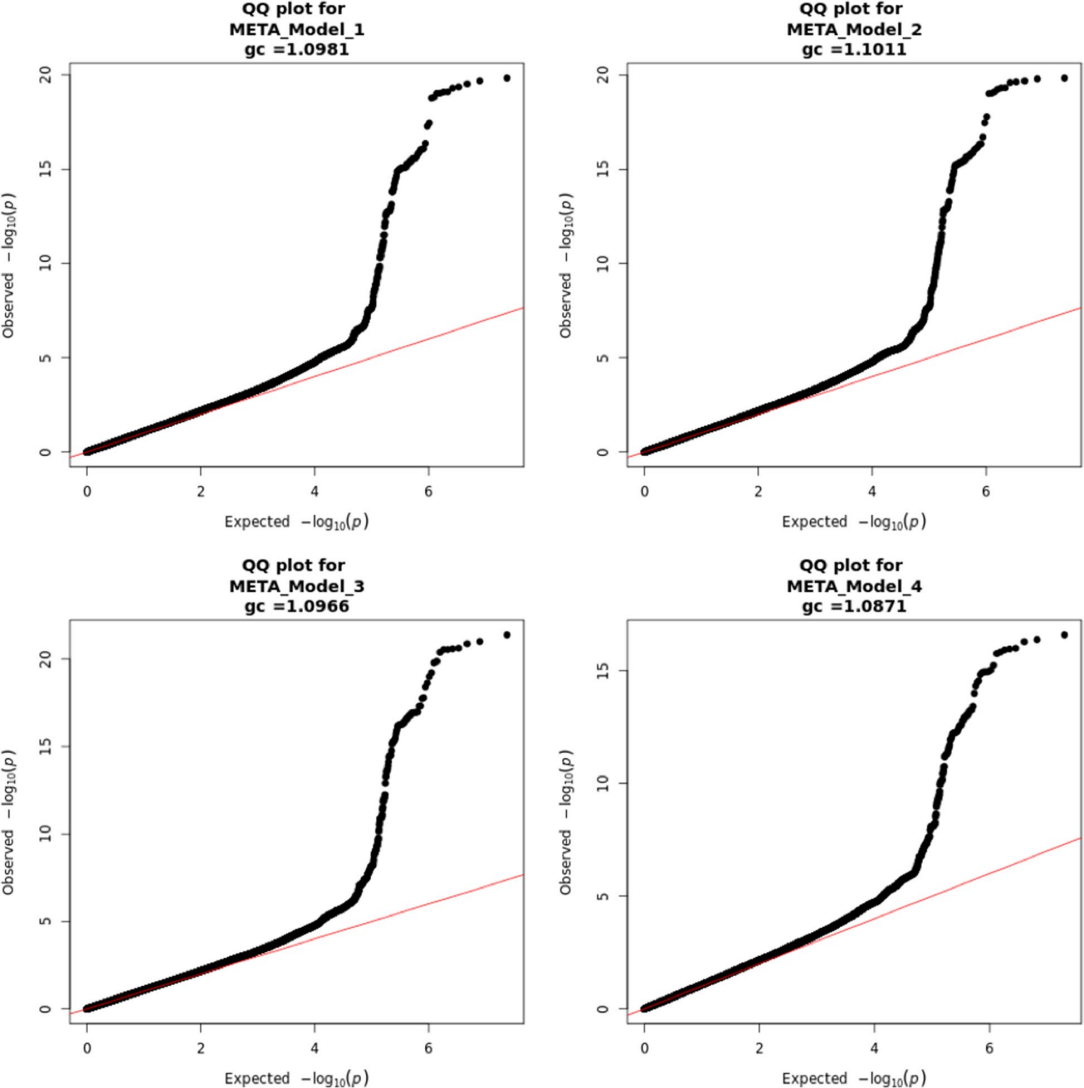
QQ-plots for all models in meta-analyses. Legends: On the y-axis the observed log10 *P*-values with the expected log10 *P*-values on the x-axis. gc = lambda genomic control

In meta-analysis of M1 9,900,019 SNPs were tested, in M2 9,897,818 and in M3 and M4 9,889,051 SNPs were tested. SNPs were tested when they passed QC in one of two datasets. In total, five SNPs on chromosomes 8, 11, 15 and 16 exceeded the genome wide significance threshold (Table 2). The top SNPs of these regions were well-imputed or genotyped and of good quality (Additional File 4: Table S1). In Figs. 1 and 2 Manhattan plots and QQ-plots with the λgc of models 1–4 are displayed.

**Table 2:** GWAS TopSNPs results of model 1, 2, 3 and 4

**Model**	**SNP**	**Chr**	**Position (bp)**	**Alleles**^**a**^		**Locus/nearest gene(s)**	**GSA GWAS**			**CytoSNP GWAS**			**Meta-analysis**		
				**Minor**	**Ref**		**MAF**	***p* value**	**B** **(SE)**	**MAF**	***p* value**	**B** **(SE)**	***p* value**	**B** **(SE)**	**Het. *p* value**
1	rs12931267	16	89,818,732	G	C	*FANCA*	0.07	2.3 × 10^−13^	0.025 (0.003)	0.07	2.1 × 10^−08^	0.026 (0.005)	3.0 × 10^−20^	0.025 (0.003)	0.89
	rs2846707	11	102,576,358	T	C	*MMP27*	0.35	4.9 × 10^−11^	-0.012 (0.002)	0.35	9.2 × 10^−05^	-0.010 (0.002)	2.7 × 10^−14^	-0.011 (0.002)	0.46
	rs2470893	15	75,019,449	T	C	*CYP1A1*	0.34	8.9 × 10^−09^	0.011 (0.002)	0.33	1.5 × 10^−05^	0.011 (0.002)	6.6 × 10^−13^	0.011 (0.002)	1.00
	rs576201050	8	18,288,053	A	G	*NAT2*	0.01	7.3 × 10^−07^	-0.044 (0.009)	0.01	1.5 × 10^−05^	-0.053 (0.012)	3.6 × 10^−11^	-0.047 (0.007)	0.55
2	rs12931267	16	89,818,732	G	C	*FANCA*	0.07	1.3 × 10^−13^	0.025 (0.003)	0.07	2.0 × 10^−08^	0.026 (0.005)	2.5 × 10^−20^	0.025 (0.003)	0.93
	rs2846707	11	102,576,358	T	C	*MMP27*	0.35	4.4 × 10^−11^	-0.012 (0.002)	0.35	9.8 × 10^−05^	-0.010 (0.002)	2.4 × 10^−14^	-0.011 (0.002)	0.45
	rs2470893	15	75,019,449	T	C	*CYP1A1*	0.34	6.7 × 10^−09^	0.011 (0.002)	0.33	1.4 × 10^−05^	0.011 (0.002)	5.4 × 10^−13^	0.011 (0.002)	0.99
	rs576201050	8	18,288,053	A	G	*NAT2*	0.01	5.8 × 10^−07^	-0.044 (0.009)	0.01	1.3 × 10^−05^	-0.053 (0.012)	2.5 × 10^−11^	-0.047 (0.007)	0.55
3	rs12931267	16	89,818,732	G	C	*FANCA*	0.07	5.8 × 10^−15^	0.026 (0.003)	0.07	1.6 × 10^−08^	0.026 (0.005)	4.3 × 10^−22^	0.026 (0.003)	0.91
	rs2846707	11	102,576,358	T	C	*MMP27*	0.35	5.7 × 10^−11^	-0.012 (0.002)	0.35	5.2 × 10^−05^	-0.011 (0.002)	2.4 × 10^−14^	-0.011 (0.002)	0.85
	rs2470893	15	75,019,449	T	C	*CYP1A1*	0.34	3.5 × 10^−05^	0.008 (0.002)	0.33	2.3 × 10^−03^	0.008 (0.002)	3.0 × 10^−7^	0.008 (0.002)	0.97
	rs576201050	8	18,288,053	A	G	*NAT2*	0.01	1.6 × 10^−07^	-0.046 (0.009)	0.01	1.8 × 10^−05^	-0.052 (0.012)	8.7 × 10^−12^	-0.048 (0.007)	0.67
4	rs12931267	16	89,818,732	G	C	*FANCA*	0.07	3.3 × 10^−11^	0.023 (0.003)	0.07	2.2 × 10^−07^	0.024 (0.005)	3.2 × 10^−17^	0.023 (0.003)	0.83
	rs2846707	11	102,576,358	T	C	*MMP27*	0.35	6.3 × 10^−11^	-0.012 (0.002)	0.35	3.8 × 10^−06^	-0.011 (0.002)	1.1 × 10^−15^	-0.012 (0.002)	0.90
	rs2470893	15	75,019,449	T	C	*CYP1A1*	0.34	2.8 × 10^−05^	0.008 (0.002)	0.33	2.8 × 10^−03^	0.007 (0.002)	2.9 × 10^−7^	0.008 (0.002)	0.92
	rs576201050	8	18,288,053	A	G	*NAT2*	0.01	1.3 × 10^−07^	-0.046 (0.009)	0.01	1.8 × 10^−05^	-0.052 (0.012)	7.7 × 10^−12^	-0.048 (0.007)	0.69

Genome-wide significance, *p* < 5 × 10^−8^

^a^Alleles are aligned to the forward strand of NCBI Build 37

Chr., chromosome; Ref., reference; MAF, minor allele frequency; Het. heterogeneity *p* value: *p* value from METAL’s *heterogeneity analysis*

Model 1 was adjusted for sex, smoking status, source of inclusion into the Lifelines cohort, age, age squared, body mass index (BMI), eGFR, rs1495741 homozygosity for the G allele and rs1495741 heterozygosity [16]. Model 2, was adjusted for all parameters of model 1 and HbA1c; Model 3, all parameters of model 2 and coffee drinking status and the number of cups of coffee per day; Model 4, Model 3 plus UV-reflectance as an additional covariate

### 16q24.3 (rs12931267)

**Fig. 3:**
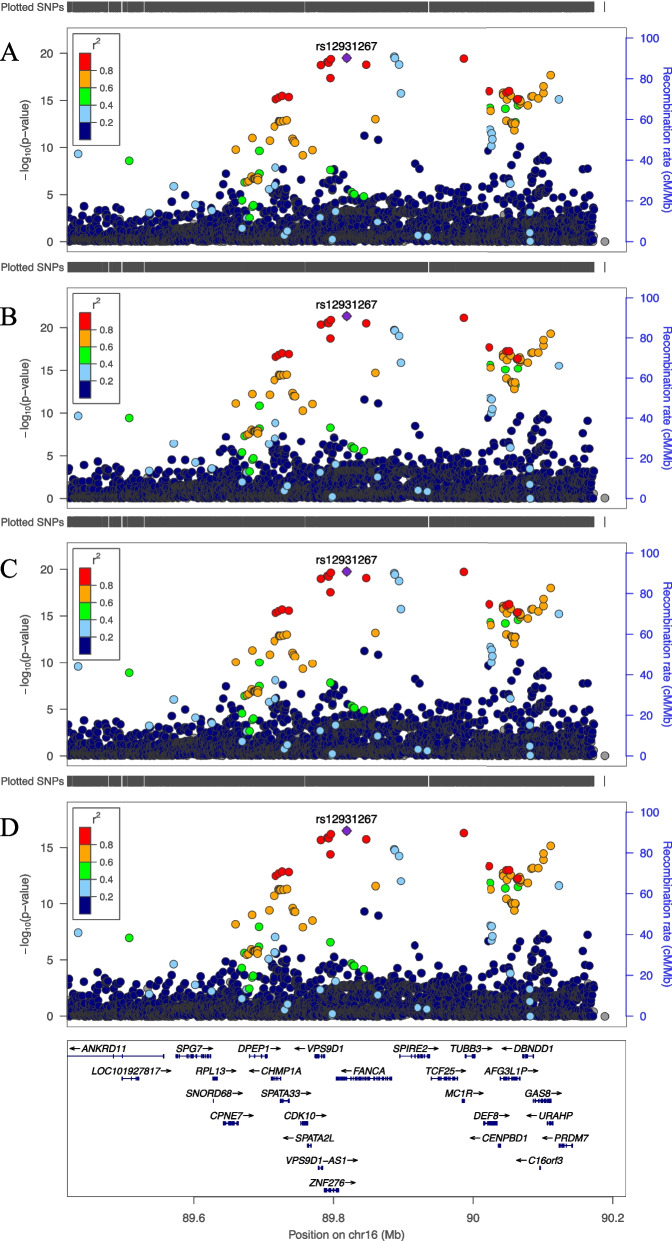
LocusZoom plots of genome wide significantly associated locus on chromosome 16. Legends: Regional plots of 400 kb surrounding rs12931267 from meta-analysis of models 1–4 (A-D, respectively). On the left y-axis the SNP P values on the x-axis their genomic positions and on the right y-axis the estimated recombination rates

The G-allele of rs12931267 on 16q24.3 (Chr16:89,818,732,C > G, build GRCh37) was associated with higher SAF in all models with the smallest *P*-value (model 1: ß = 0.025, SE = 0.003, *P* = 3.0 × 10^–20^). The SNP is in intron 30 of *FANCA* (NM_000135.4), near *MC1R* (Fig. 3). The beta and direction of the effect was similar across all models, adding SR in model 4 led to a slight attenuation of the SNP-effect. The SNP explains 0.11% of the SAF variance in the GSA data in model 4. There was no significant interaction of rs12931267 with SR on SAF *P* = 0.19, Additional File 5: Table S2.

Conditional analysis in model 4 where rs12931267 was included additively as a covariate to the model revealed another SNP in this region exceeding the genome-wide significance threshold: rs3764257 (Chr16:89,800,887,C > G, build GRCh37, β = -0.010, SE = 0.0016, *P* = 3.8 × 10^–10^). rs3764257 is 17.8 kb upstream from rs12931267, these SNPs are on the same haplotype (D’ = 0.998, r^2^ = 0.03) but there was no significant interaction between the two SNPs (*P* = 0.07). The minor C-allele of rs3764257, with allele frequency of 28%, was associated with lower SAF. The SNP is an intronic variant in *ZNF276*. This variant explains 0.02% of the SAF variance in the GSA data in model 4.

Additional conditional analysis adjusted for both rs12931267 and rs3764257 coded additively in model 1 did not reveal other independently associated genome-wide significant loci in the region.

### 11q22.2 (rs2846707)

**Fig. 4:**
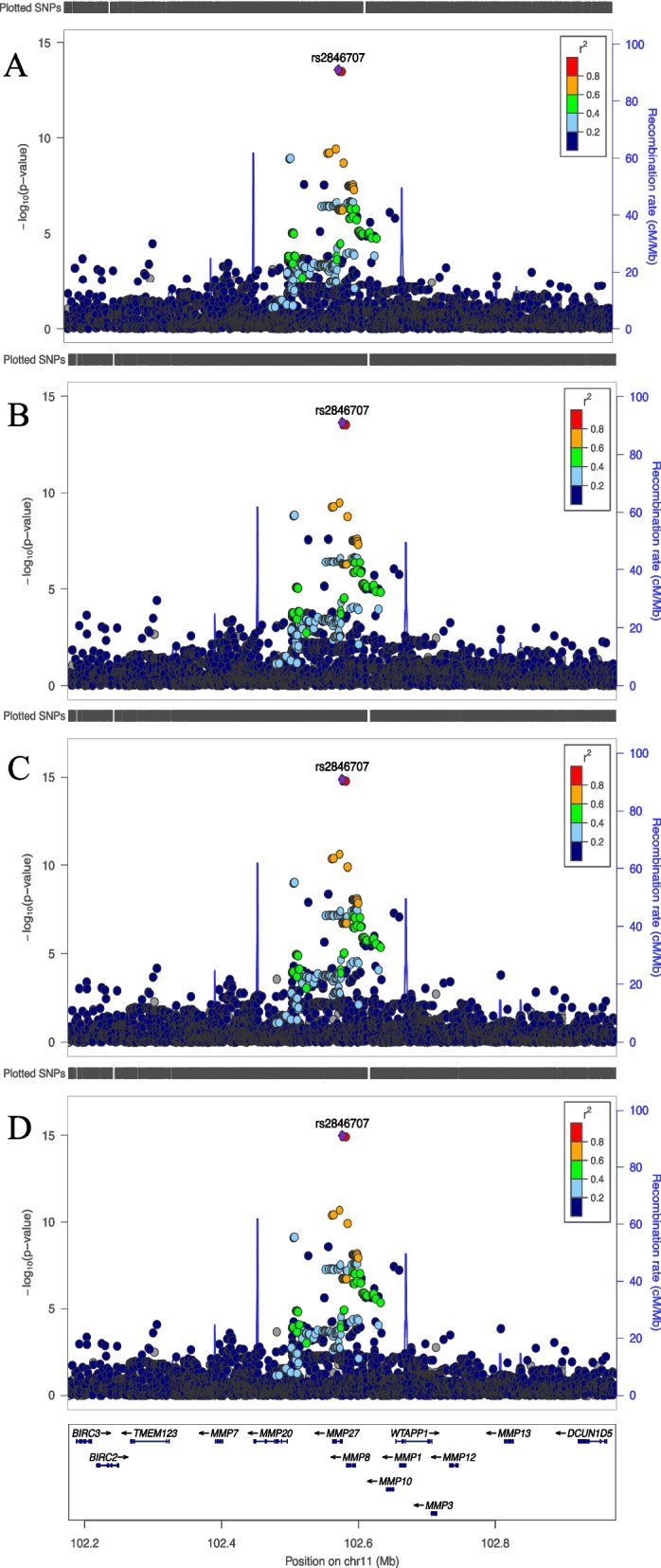
LocusZoom plots of genome wide significantly associated locus on chromosome 11. Legends: Regional plots of 400 kb surrounding rs2846707 from meta-analysis of models 1–4 (A-D, respectively). On the left y-axis the SNP P values on the x-axis their genomic positions and on the right y-axis the estimated recombination rates

rs2846707 (Chr11:102,576,358,C > T) is a well-imputed SNP, and the alternate T-allele results in a Met30Val missense variant in *MMP27* exon 1 (NM_022122.3). The presence of the T-allele was associated with lower SAF. The SNP-effect was similar across the different models (Fig. 4). 0.11% of the variance was explained by this variant in model 4 of the GSA data.

There was no significant association of rs2846707 with SR (*P* = 0.66).

### 15q24.1 (rs2470893)

**Fig. 5:**
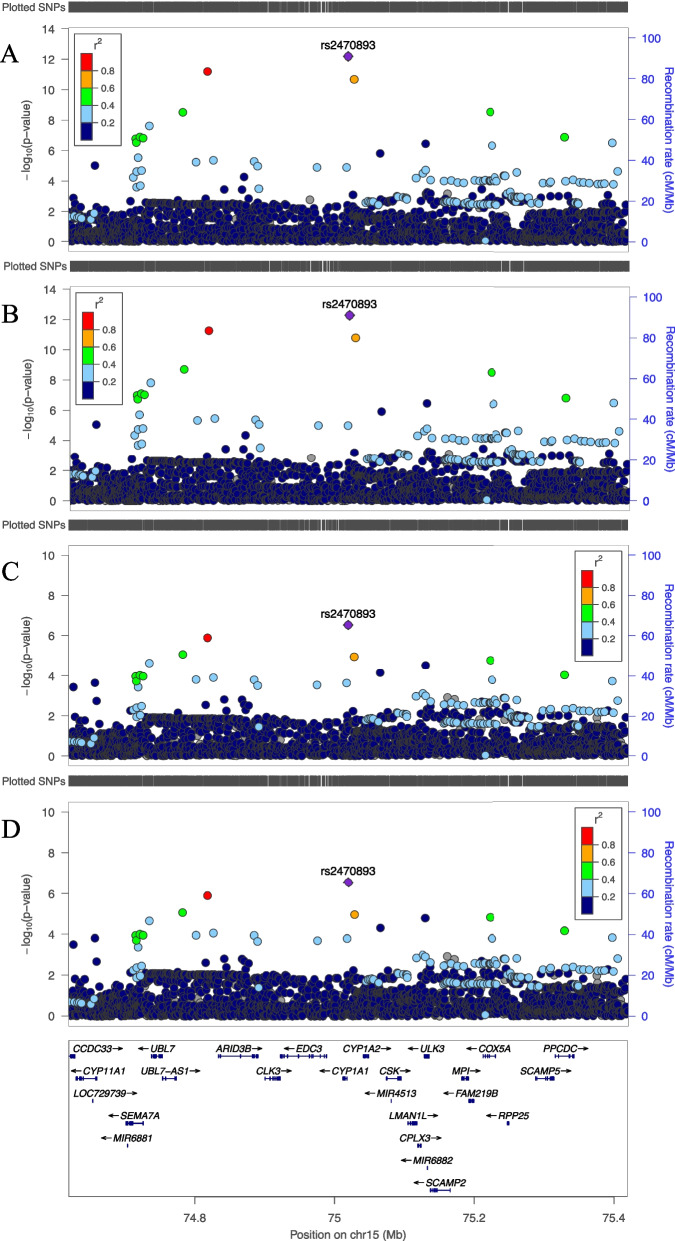
LocusZoom plots of genome wide significantly associated locus on chromosome 15. Legends: Regional plots of 400 kb surrounding rs2470893 from meta-analysis of models 1–4 (A-D, respectively). On the left y-axis the SNP P values on the x-axis their genomic positions and on the right y-axis the estimated recombination rates

The T-allele of rs2470893 (Chr15:75,019,449,C > T), in intergenic region on chromosome 15 located between *CYP1A1* and *CYP1A2* (Fig. 5) was associated with higher SAF (β = 0.011, SE = 0.002, *P* = 6.6 × 10^–13^ in model 1). The SNP-effect was attenuated in Model 3 where coffee intake was included as a covariate to the model (β = 0.008, SE = 0.002, *P* = 3.0 × 10^–7^). In model 4 of the GSA data the SNP explained 0.04% of the variance of SAF.

The interaction-term of rs2470893 with daily coffee consumption was not significant for SAF (*P* = 0.33). Also, the interaction-term of the SNP with eGFR was not associated with SAF (*P* = 0.88, Additional File 6: Table S3).

### 8p22 (rs576201050)

**Fig. 6:**
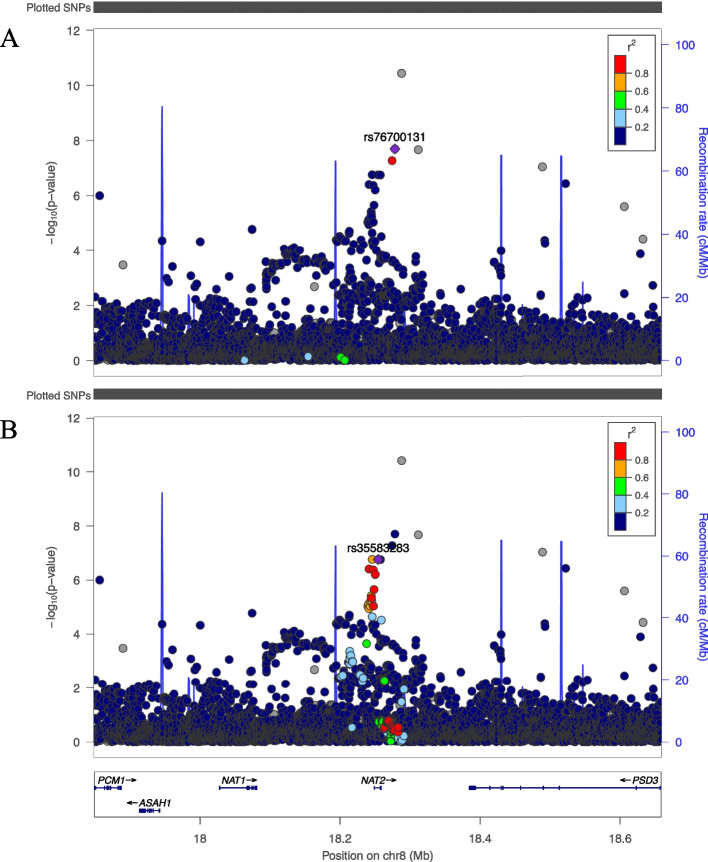
LocusZoom plots of genome wide significantly associated locus on chromosome 8. Legends: Regional plots of 400 kb surrounding NAT2 from meta-analysis of model 1, A: with r^2^ values for the SNPs surrounding rs76700131 and B: with r^2^ values for the SNPs surrounding rs35583283. On the left y-axis the SNP P values on the x-axis their genomic positions and on the right y-axis the estimated recombination rates. The gray dots indicate that the LD scores cannot be visualized because the SNP was not present in the reference dataset

rs576201050 (Chr8:18,288,053,G > A) is an independent locus situated approximately 30 kb downstream from *NAT2* on chromosome 8, which was significantly associated with SAF (Fig. 6). The minor allele A, with frequency of 1%, was associated with a lower SAF value (ß = -0.047, SE = 0.007, *P* = 3.6 × 10^−11^). The added variance explained with this SNP when added in model 4 of the GSA data was 0.07%.

Interaction term of rs576201050 and rs1495741 (the NAT2-acetylator tagging SNP) was significantly associated with SAF in the GSA data (Additional File 7: Table S4). The SNPs are in LD (D' = 1 and r^2^ = 0.003).

### Estimation of SAF variance

Of all models, GWAS model 4 explained the highest percentage of the SAF variance, 48.54%. Adding all five newly identified SNPs to the linear regression model increased the adjusted r^2^ to 48.96%. Final GWAS adjusted for all covariates in M4 including all 5 newly identified SNPs did not reveal additional loci associated with SAF.

The association of the covariates with the different models for SAF in the GSA and CytoSNP datasets are displayed in Tables 3 and 4, respectively. Covariates in the regression model of the GSA data explained 46–49% of the SAF variance while the same covariates in the models explain 37–39% of the SAF variance in the CytoSNP data. Adding more covariates in models 2–4 does not drastically improve adjusted r^2^. The total variance explained for SAF is increased by 2.4% in the GSA data and 3.6% in the CytoSNP data when all genetic variants (including *NAT2*) are added to the model, compared with the model only adjusted for the known environmental and lifestyle determinants.

**Table 3:** Linear regression models for lnSAF in R (GSA)

	**Model 1**			**Model 2**			**Model 3**			**Model 4**		
**Covariate**	**Beta**	**SE**	**P-value**	**Beta**	**SE**	**P-value**	**Beta**	**SE**	**P-value**	**Beta**	**SE**	**P-value**
**Age**	1.5 × 10^–02^	6.0 × 10^–04^	< 2 × 10^–16^	1.5 × 10^–02^	6.0 × 10^–04^	< 2 × 10^–16^	1.0 × 10^–02^	6.2 × 10^–04^	< 2 × 10^–16^	1.0 × 10^–02^	6.1 × 10^–04^	< 2 × 10^–16^
**Age squared**	-4.9 × 10^–05^	6.8 × 10^–06^	3.0 × 10^–13^	-4.9 × 10^–05^	6.8 × 10^–06^	4.4 × 10^–13^	-9.2 × 10^–06^	6.8 × 10^–06^	1.8 × 10^–01^	-7.1 × 10^–06^	6.8 × 10^–06^	3.0 × 10^–01^
**Male sex**	2.5 × 10^–02^	2.6 × 10^–03^	< 2 × 10^–16^	2.5 × 10^–02^	2.6 × 10^–03^	< 2 × 10^–16^	6.7 × 10^–03^	2.6 × 10^–03^	1.1 × 10^–02^	9.8 × 10^–03^	2.6 × 10^–03^	1.9 × 10^–04^
**Smoking status**												
** non-smoker**	Ref	-	-	Ref	-	-	Ref	-	-	Ref	-	-
** previous smoker**	3.9 × 10^–02^	3.5 × 10^–03^	< 2 × 10^–16^	3.9 × 10^–02^	3.5 × 10^–03^	< 2 × 10^–16^	2.8 × 10^–02^	3.4 × 10^–03^	9.0 × 10^–16^	2.9 × 10^–02^	3.4 × 10^–03^	< 2 × 10^–16^
** current smoker**	8.3 × 10^–02^	3.3 × 10^–03^	< 2 × 10^–16^	8.2 × 10^–02^	3.3 × 10^–03^	< 2 × 10^–16^	6.2 × 10^–02^	3.3 × 10^–03^	< 2 × 10^–16^	6.4 × 10^–02^	3.3 × 10^–03^	< 2 × 10^–16^
** BMI**	2.7 × 10^–03^	3.2 × 10^–04^	< 2 × 10^–16^	2.6 × 10^–03^	3.2 × 10^–04^	1.0 × 10^–15^	2.3 × 10^–03^	3.1 × 10^–04^	3.7 × 10^–13^	1.8 × 10^–03^	3.2 × 10^–04^	1.4 × 10^–08^
** eGFR**	-4.9 × 10^–04^	1.1 × 10^–04^	5.0 × 10^–06^	-4.9 × 10^–04^	1.1 × 10^–04^	7.4 × 10^–06^	-4.7 × 10^–04^	1.1 × 10^–04^	8.3 × 10^–06^	-5.3 × 10^–04^	1.1 × 10^–04^	7.2 × 10^–07^
** rs1495741 copies G-allele**	-3.7 × 10^–02^	2.8 × 10^–03^	< 2 × 10^–16^	-3.7 × 10^–02^	2.8 × 10^–03^	< 2 × 10^–16^	-3.7 × 10^–02^	2.8 × 10^–03^	< 2 × 10^–16^	-3.8 × 10^–02^	2.8 × 10^–03^	< 2 × 10^–16^
** rs1495741 heterozygosity**	-2.4 × 10^–02^	3.5 × 10^–03^	2.1 × 10^–11^	-2.4 × 10^–02^	3.5 × 10^–03^	2.2 × 10^–11^	-2.4 × 10^–02^	3.4 × 10^–03^	7.6 × 10^–12^	-2.4 × 10^–02^	3.4 × 10^–03^	7.9 × 10^–12^
**Inclusion method**												
** Family doctor**	Ref	-	-	Ref	-	-	Ref	-	-	Ref	-	-
** Included family members**	-7.5 × 10^–03^	3.1 × 10^–03^	1.4 × 10^–02^	-7.6 × 10^–03^	3.1 × 10^–03^	1.2 × 10^–02^	-6.2 × 10^–03^	3.0 × 10^–03^	3.8 × 10^–02^	-6.3 × 10^–03^	3.0 × 10^–03^	3.4 × 10^–02^
** Self-administrated**	-1.5 × 10^–02^	4.0 × 10^–03^	1.5 × 10^–04^	-1.5 × 10^–02^	4.0 × 10^–03^	1.3 × 10^–04^	-1.3 × 10^–02^	3.9 × 10^–03^	1.2 × 10^–03^	-1.2 × 10^–02^	3.9 × 10^–03^	1.7 × 10^–03^
** HbA1c**	-	-	-	1.6 × 10^–03^	4.4 × 10^–04^	3.0 × 10^–04^	1.5 × 10^–03^	4.3 × 10^–04^	3.1 × 10^–04^	1.5 × 10^–03^	4.3 × 10^–04^	5.7 × 10^–04^
** Coffee drinking status**	-	-	-	-	-	-	1.2 × 10^–02^	4.5 × 10^–03^	9.2 × 10^–03^	1.2 × 10^–02^	4.4 × 10^–03^	6.0 × 10^–03^
** cups per day**	-	-	-	-	-	-	1.6 × 10^–02^	7.1 × 10^–04^	< 2 × 10^–16^	1.6 × 10^–02^	7.1 × 10^–04^	< 2 × 10^–16^
** Reflectance**	-	-	-	-	-	-	-	-	-	1.9 × 10^–01^	1.9 × 10^–02^	< 2 × 10^–16^
** Adjusted R-squared**	0.46			0.46			0.48			0.49

**Table 4:** Linear regression models for lnSAF in R (CytoSNP)

	**Model 1**			**Model 2**			**Model 3**			**Model 4**		
**Covariate**	**Beta**	**SE**	**P-value**	**Beta**	**SE**	**P-value**	**Beta**	**SE**	**P-value**	**Beta**	**SE**	**P-value**
**Age**	1.7 × 10^–02^	1.0 × 10^–03^	< 2 × 10^–16^	1.7 × 10^–02^	1.0 × 10^–03^	< 2 × 10^–16^	1.3 × 10^–02^	1.1 × 10^–03^	< 2 × 10^–16^	1.3 × 10^–02^	1.1 × 10^–03^	< 2 × 10^–16^
**Age squared**	-7.1 × 10^–05^	1.0 × 10^–05^	3.6 × 10^–12^	-7.0 × 10^–05^	1.0 × 10^–05^	6.9 × 10^–12^	-3.1 × 10^–05^	1.1 × 10^–05^	3.6 × 10^–03^	-3.2 × 10^–05^	1.1 × 10^–05^	2.8 × 10^–03^
**Male sex**	2.4 × 10^–02^	3.4 × 10^–03^	1.6 × 10^–12^	2.4 × 10^–02^	3.4 × 10^–03^	2.1 × 10^–12^	6.8 × 10^–03^	3.5 × 10^–03^	5.3 × 10^–02^	8.8 × 10^–03^	3.5 × 10^–03^	1.3 × 10^–02^
**Smoking status**												
** non-smoker**	Ref	-	-	Ref	-	-	Ref	-	-	Ref	-	-
** previous smoker**	3.6 × 10^–02^	4.6 × 10^–03^	6.7 × 10^–15^	3.6 × 10^–02^	4.6 × 10^–03^	6.4 × 10^–15^	2.7 × 10^–02^	4.5 × 10^–03^	1.5 × 10^–09^	2.8 × 10^–02^	4.5 × 10^–03^	5.7 × 10^–10^
** current smoker**	1.0 × 10^–01^	4.3 × 10^–03^	< 2 × 10^–16^	1.0 × 10^–01^	4.3 × 10^–03^	< 2 × 10^–16^	8.1 × 10^–02^	4.4 × 10^–03^	< 2 × 10^–16^	8.2 × 10^–02^	4.4 × 10^–03^	< 2 × 10^–16^
** BMI**	2.0 × 10^–03^	4.0 × 10^–04^	7.9 × 10^–07^	1.8 × 10^–03^	4.1 × 10^–04^	8.0 × 10^–06^	1.7 × 10^–03^	4.1 × 10^–04^	2.3 × 10^–05^	1.4 × 10^–03^	4.1 × 10^–04^	5.8 × 10^–04^
** eGFR**	-6.4 × 10^–04^	1.5 × 10^–04^	1.3 × 10^–05^	-6.4 × 10^–04^	1.5 × 10^–04^	1.3 × 10^–05^	-6.2 × 10^–04^	1.4 × 10^–04^	1.9 × 10^–05^	-6.3 × 10^–04^	1.4 × 10^–04^	1.2 × 10^–05^
** rs1495741 copies G-allele**	-3.9 × 10^–02^	3.9 × 10^–03^	< 2 × 10^–16^	-3.9 × 10^–02^	3.9 × 10^–03^	< 2 × 10^–16^	-4.0 × 10^–02^	3.9 × 10^–03^	< 2 × 10^–16^	-4.0 × 10^–02^	3.9 × 10^–03^	< 2 × 10^–16^
** rs1495741 heterozygosity**	-3.3 × 10^–02^	4.8 × 10^–03^	5.4 × 10^–12^	-3.4 × 10^–02^	4.8 × 10^–03^	3.8 × 10^–12^	-3.3 × 10^–02^	4.8 × 10^–03^	6.2 × 10^–12^	-3.3 × 10^–02^	4.8 × 10^–03^	4.1 × 10^–12^
**Inclusion method**												
** Family doctor**	Ref	-	-	Ref	-	-	Ref	-	-	Ref	-	-
** Included family members**	-1.7 × 10^–04^	5.0 × 10^–03^	9.7 × 10^–01^	-8.7 × 10^–04^	5.0 × 10^–03^	8.6 × 10^–01^	-1.3 × 10^–03^	5.0 × 10^–03^	7.9 × 10^–01^	-9.2 × 10^–04^	5.0 × 10^–03^	8.5 × 10^–01^
** Self-administrated**	-5.8 × 10^–03^	6.8 × 10^–03^	3.9 × 10^–01^	-6.6 × 10^–03^	6.8 × 10^–03^	3.3 × 10^–01^	-5.8 × 10^–03^	6.7 × 10^–03^	3.8 × 10^–01^	-5.8 × 10^–03^	6.7 × 10^–03^	3.8 × 10^–01^
** HbA1c**	-	-	-	1.3 × 10^–03^	5.8 × 10^–04^	2.7 × 10^–02^	1.7 × 10^–03^	5.7 × 10^–04^	3.5 × 10^–03^	1.7 × 10^–03^	5.7 × 10^–04^	3.8 × 10^–03^
** Coffee drinking status**	-	-	-	-	-	-	2.0 × 10^–02^	7.6 × 10^–03^	7.6 × 10^–03^	2.1 × 10^–02^	7.6 × 10^–03^	6.8 × 10^–03^
** cups per day**	-	-	-	-	-	-	1.5 × 10^–02^	8.9 × 10^–04^	< 2 × 10^–16^	1.5 × 10^–02^	8.9 × 10^–04^	< 2 × 10^–16^
** Reflectance**	-	-	-	-	-	-	-	-	-	1.1 × 10^–01^	2.8 × 10^–02^	1.6 × 10^–04^
** Adjusted R-squared**	0.37			0.37			0.39			0.39		

### Replication of earlier reported associations

In addition to rs1495741 (NAT2-acetylator tagging SNP), rs7533564 (Chr1:78,825,912, C < T) was associated with SIF (Skin Intrinsic Fluorescence; a comparable measurement to SAF measured with the SCOUT DS device) in a previous study in type 1 diabetes individuals [17]. This SNP was not associated with SAF in Lifelines (β = -0.001, SE = 0.005, *P* = 0.82, MAF 2.1% in meta-analysis of model 1).

### GWAS of skin reflectance

The results of the GWAS of SR are summarized in Additional File 8: Table S5, Additional File 9: Table S6 and Additional File 10: Figure S2. In total, we identified 21 independent signals which were associated with SR. The strongest association was observed with rs35096708 on chromosome 16 near *MC1R*, intronic to *CPNE7*. Conditional analyses revealed that there were fourteen independent SNPs associated with SR in this region (Additional File 9: Table S6). All other loci were independently associated with SR but had no genome-wide significant association with SAF. All SNPs for SAF were tested for association with SR (Additional File 11: Table S7), and in addition to rs12931267 and rs3764257, rs1495741 (NAT2-acetylator tagging SNP) was nominally associated with SR with the opposite direction of effect.

### SNPs for coffee consumption and SAF

As higher daily coffee consumption is associated with higher SAF levels [32], and the association of rs2470893 in the current study was attenuated when coffee intake was included as a covariate in our models. We decided to test all known SNPs associated with coffee consumption for association with SAF [33]. The results of these post-hoc evaluations are shown in Additional File 12: Table S8. In addition to rs2470893 near *CYP1A1/2*, rs10865548, rs4410790, rs73073176, rs1057868 and rs2330783 were nominally associated with SAF with the same directions of effect.

### Expression quantitative trait loci (cis-eQTLs)

In Additional Files 13,14,15,16: Supplementary Tables S9-12 cis-eQTLs of the top associated SNPs for SAF are described [18]. Four out of five novel identified SNPs for SAF were cis-eQTLs in GTEx (v8) and were associated with expression of various genes within the chromosomal region and in various tissue. The SNPs near *NAT2* i.e. rs1495741 and rs576201050 were not cis-eQTLs.

## Discussion

In this large group of individuals without diabetes of Western European descent, we estimated the heritability of SAF to be 33%, taking into account the known (in part environmental) factors which may influence SAF levels. We identified four novel loci with 5 SNPs, one on chromosomes 11 and 15, two on chromosome 16, and one locus on chromosome 8 near the previously identified locus *NAT2,* that were associated with SAF.

Most of the studies on SAF have reported on environmental factors associated with this measurement [32, 34]. SAF increases with ageing, and is elevated in people with type 2 diabetes compared with age-matched controls [11, 35]. It was demonstrated that SAF is already elevated in people without diabetes but with metabolic syndrome, and is associated with some of its individual components [36]. Furthermore, SAF is strongly related to current smoking status as well as smoking history, coffee consumption, and renal function [32]. In the general population SAF is a strong predictor of the development of cardiovascular disease and mortality [37]. In addition, higher SAF levels predicted a higher risk of development of type 2 diabetes, and algorithms have been developed to predict individuals with the highest risk of developing type 2 diabetes based on age, BMI, and SAF levels [37]. SAF reflects fluorescent properties present in skin and skin collagen half-life is about 15 years [38]. As a result, SAF is a marker for cumulative accumulation over a much longer period of time than for instance haemoglobin A1c [39]. Compared with excreted molecules or markers, SAF may therefore be a more appropriate tool for screening for diseases that develop over time.

Because of the growing interest to use SAF to screen for type 2 diabetes, diabetes complication risks, cardiovascular disease and mortality [12, 37], more information on possible genetic determinants of SAF is warranted, especially because studies of serum AGEs have estimated heritability of 63–74% [14, 15]. Understanding the genetics of a quantitative trait can improve performance of the tool. Unfortunately, there are only a few genetic studies of SAF [16, 17].

Our previous study reported the genetic association between a SNP in *NAT2* and SAF, in a first set of approximately 9000 participants of the Lifelines Cohort Study, that overlaps partly with those reported in the CytoSNP cohort here [16]. We also reported the association of a locus on chromosome 1 and SIF, but this association was only observed in individuals with type 1 diabetes [14]. The present study has combined the earlier dataset of the Lifelines Cohort Study, and added a new set of over 17,000 individuals genotyped recently on the Illumina Infinium Global Screening Array-24 (GSA) version 1.

In meta-GWAS, we identified five additional loci which are associated with SAF in individuals without diabetes.

The association of the locus on chromosome 16 was the most significantly associated with SAF after *NAT2*. rs12931267 was significantly associated in all GWAS models, however the SNP-effect was slightly attenuated in model 4 where SR was added as a covariate. The SNP is near *MC1R*, in which multiple independent coding variants have been associated with skin colour and pigmentation traits [40, 41]. In GWAS of SR measures from the same individuals we identified multiple genome wide significant signals in the *MC1R* region as well. Through internal software in the AGE Reader, SAF is adjusted for variation in skin colour by SR [22]. However, in multivariable models SR and SAF are still correlated. This association is complex and requires further investigation.

On chromosome 11, rs2846707 which results in a Met30Val missense variant in *MMP27* exon 1 was associated with SAF. The pathologic significance of the association could be by the altering of an amino acid, however the SNP is also a cis-eQTL and associates with the expression level of several nearby genes. It is not clear whether there is allelic imbalance as the functional impact of this specific variant in *MMP27* has not been investigated. Previous research did demonstrate an interplay between matrix metalloproteinases (MMPs) and AGEs, specifically because of the known collagen degrading activity of MMPs [42–44]. There was no association of this SNP with SR. In PheWeb, comprising the genetic (TOPMed imputed) and clinical information from the White British participants of the UK Biobank, the trait with the smallest *P*-value for rs2846707 was ‘Diffuse diseases of connective tissue’ (p = 0.002) and the second most associated trait was ‘Diabetic retinopathy’ (p = 0.002) [45, 46].

The effect of rs2470893 on chromosome 15 was attenuated when coffee intake -an important environmental factor influencing SAF- was included as a covariate. It can be postulated that some SNPs which are associated with daily coffee consumption may also be associated with SAF levels, based on the fact that higher coffee consumption is associated with higher SAF [32, 34]. Indeed, in our post-hoc evaluation, we found that multiple SNPs associated with coffee consumption were also nominally associated with SAF with similar direction of the effect (additional File 12, Table S8). A variety of factors potentially resulting from the roasting process of coffee beans (Maillard Reaction) may be involved in the association of increased coffee consumption and higher SAF, examples are the loss of chlorogenic acid, the presence of melanoidins or caffeine [32, 34, 47]). The relative contribution of each of these fluorescent factors, and potentially others, on SAF is unknown.

We identified an additional independent locus in the *NAT2* region that was associated with SAF, which underlines the robust association of *NAT2* variants with SAF. Previous research identified that *NAT2* is also associated with insulin resistance [48]. This shared association of insulin resistance as well as SAF with *NAT2* may, in part, be explanatory for the predictive value of SAF for type 2 diabetes development.

We examined all SNPs in the GTEx project and found that rs12931267, rs2846707, rs2470893 and rs3764257 are all cis-eQTLs for various nearby genes. However, if the SNP-effect on SAF is explained by the association of the SNP with gene-expression, requires further investigation. For rs1495741 and rs576201050 we found no cis-eQTLs effects in GTEx. This may be caused by low minor allele frequency of rs576201050, since cis-eQTLs are only described for variants with MAF > 1% [49]. Since rs1495741 is a tag-SNP for the acetylator phenotype which well explains the association with SAF, it is reasonable that the association is not mediated through cis-eQTL effects.

All newly identified loci explain a small percentage (below 1%) of the variance in SAF. In combination with the previously reported *NAT2* SNP they explain less than 5% of the total SAF variation. However, insight into the genetic factors associated with SAF variation does provide more knowledge of the complex pathology of formation and accumulation of AGEs. As the estimated genetic heritability of SAF was 33%, the majority of genetic variants that explain SAF variability remain undiscovered. It seems reasonable that numerous SNPs each explain a small fraction of SAF variation, which could be identified by larger GWAS studies to increase power for detection of loci with even smaller effect sizes.

We also evaluated genome-wide signals for skin reflectance, an additional measure from the AGE Reader. SR is important for the proper measurement of SAF. Using the current AGE Reader only SAF values can be reliably measured when SR measurements are above 6%. As mentioned, SR is a proxy for skin colour or pigmentary phenotype. All genetic regions associated with SR have indeed earlier been shown to be associated with skin colour traits and perceived age, including *SLC45A2*, *IRF4*, *BNC2*, *TYR*, *HERC2*, *MC1R* and *RALY/ASIP* [41, 50]. Furthermore, variants in the *MC1R* region have been shown to be strongly associated with higher susceptibility of cutaneous melanoma [13, 51]. In our heritability analyses we found that SAF and SR have some shared heritability. Part of this shared heritability can be explained by the shared clinical determinants of the two traits; i.e. kidney function, coffee consumption, smoking behaviour and BMI. This explains why the shared heritability between SAF and SR is higher in the model not adjusted for any covariates compared to the model adjusted for covariates, 33% and 24% respectively. The remaining shared heritability of the SAF and SR cannot be explained in the current study, it may depend on specific skin structures associated with both SR as well as SAF, this requires further investigation.

Limitations of the current study are the fact that we used two different genotyping arrays on different subjects and we used HRC imputed data while the TOPMed panel comprises a larger reference panel and results in more imputed SNPs as well as better quality imputation [45]. However, for imputation we are dependent on the Lifelines research group who have not yet released the TOPMed imputed Lifelines data. Another limitation is that we only analyzed autosomal SNPs while there is a considerable possibility that some of the SAF variance is explained by variation on the sex chromosomes. Finally, our study comprises only white individuals from European descent and the results are therefore not generalizable to the non-white population.

## Conclusions

The estimated heritability of SAF is 33%, considering the known factors which may influence SAF levels. We identified five novel loci which are associated with SAF in the non-diabetes population. Together with the earlier reported *NAT2* polymorphism these SNPs explain less than 5% of the SAF variance in the non-diabetes population.

## Supplementary Information

**Additional file 1.** 

**Additional file 2.** 

**Additional file 3.**

**Additional file 4.** 

**Additional file 5.** 

**Additional file 6.** 

**Additional file 7.** 

**Additional file 8.** 

**Additional file 9.** 

**Additional file 10.** 

**Additional file 11.** 

**Additional file 12.** 

**Additional file 13.** 

**Additional file 14.** 

**Additional file 15.** 

**Additional file 16.** 

## Data Availability

The manuscript is based on data from the Lifelines Cohort Study. Lifelines adheres to standards for data availability, and allows access for reproducibility of the study results. The data catalogue is publicly accessible at www.lifelines.nl. The dataset supporting the conclusions of this article is available through the Lifelines organization, and all international researchers can apply for data access at the Lifelines research office (research@lifelines.nl). For data access, a fee is required.

## Abbreviations

AGE: Advanced glycation endproducts
AU: Arbitrary units
BMI: Body mass index
CVD: Cardiovascular disease
eGFR: Estimated glomerular filtration rate
eQTL: Expression quantitative trait loci
LD: Linkage disequilibrium
GC: Genomic control
GSA: Infinium Global Screening Array-24
GWAS: Genome-wide association studies
HbA1c: Glycated haemoglobin
HRC: Haplotype reference consortium
HWE: Hardy-Weinberg Equilibrium
IQR: Interquartile range
SAF: Skin autofluorescence
SIF: Skin intrinsic fluorescence
SNP: Singe Nucleotide Polymorphism
SR: Skin reflectance
T2D: Type 2 diabetes
