# Supplementary material for: Genome-wide association study identifies novel loci associated with skin autofluorescence in individuals without diabetes
Source: BMC Genomics. 2022 Dec 19;23:840. doi: 10.1186/s12864-022-09062-x (PMC9764523; doi:10.1186/s12864-022-09062-x)
Supplement: Supplementary file 1 — Additional file 1. [file 12864_2022_9062_MOESM1_ESM.pdf]

Additional File 1: Supplementary Methods.

About Lifelines

Lifelines is a multi-disciplinary prospective population-based cohort study examining in a unique three-generation design the health and health-related behaviours of 167,729 persons living in the North of The Netherlands. It employs a broad range of investigative procedures in assessing the biomedical, socio-demographic, behavioural, physical and psychological factors which contribute to the health and disease of the general population, with a special focus on multi-morbidity and complex genetics. Subjects were recruited into the Lifelines cohort either through their family physician, as a family member (spouse, parent or child) of an individual who was already included in the study, or by voluntary self-registration.

Two subgroups from the Lifelines cohort were genotyped. The first subgroup was a random sample of 15,422 individuals who were genotyped on the Illumina CytoSNP 12v2 array. In a later phase, a second subgroup of 36,339 individuals was genotyped with the Illumina Infinium Global Screening array-24 (GSA) version 1 chip. After quality control GWAS was performed on N=9957 and N=17297 individuals in Model 1 from the CytoSNP and GSA cohort, respectively (Additional file 2: Figure S1).
