# Supplementary figures and images for: Genome-wide association study identifies novel loci associated with skin autofluorescence in individuals without diabetes
Source: BMC Genomics. 2022 Dec 19;23:840. doi: 10.1186/s12864-022-09062-x (PMC9764523; doi:10.1186/s12864-022-09062-x)

## Additional File 2: Figure S1

### Flowchart of subjects included in final analyses.

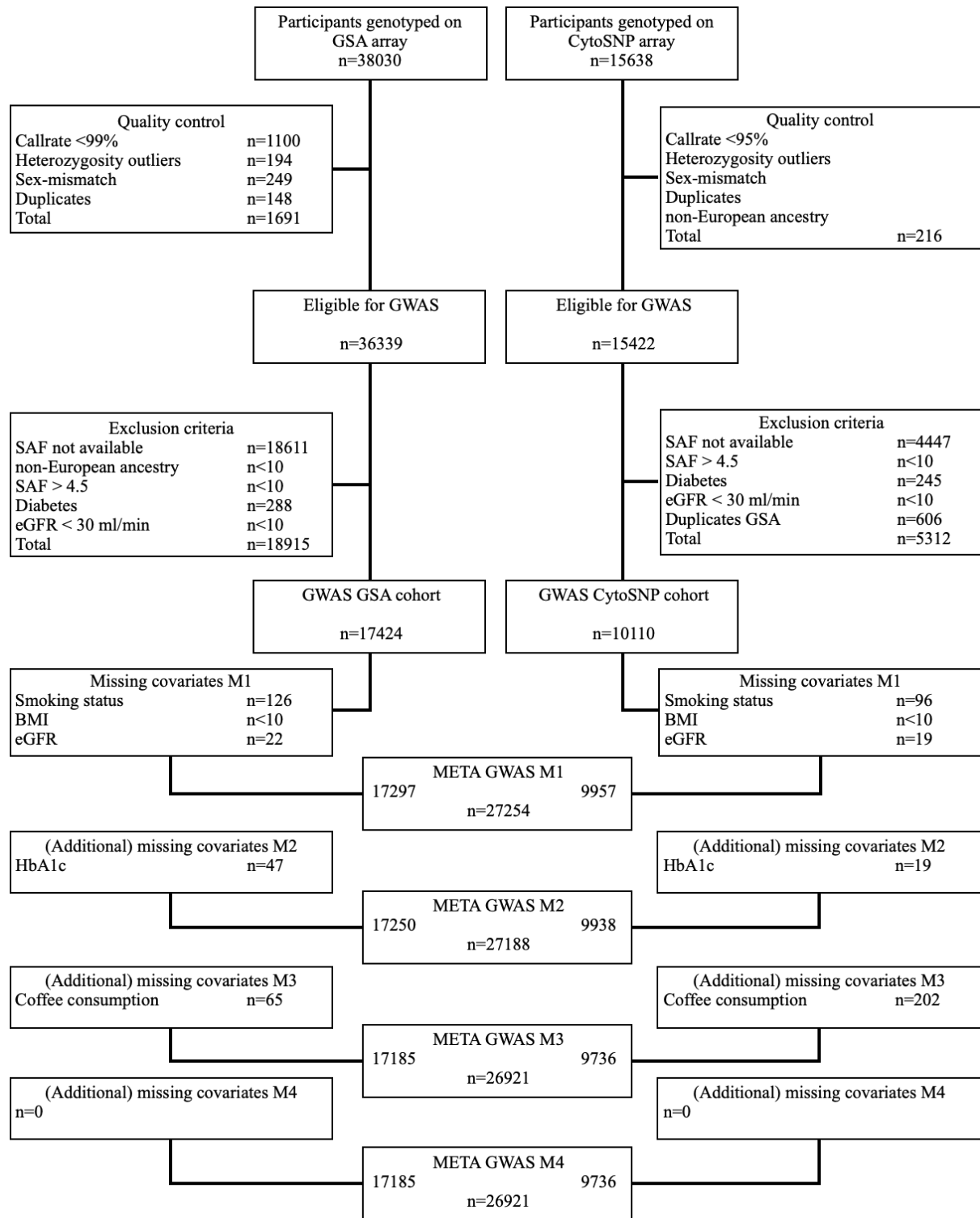

Supplement: Supplementary file 2 — Additional file 2. [file 12864_2022_9062_MOESM2_ESM.pdf]
