# Supplementary material for: Genome-wide association study identifies novel loci associated with skin autofluorescence in individuals without diabetes
Source: BMC Genomics. 2022 Dec 19;23:840. doi: 10.1186/s12864-022-09062-x (PMC9764523; doi:10.1186/s12864-022-09062-x)
Supplement: Supplementary file 3 — Additional file 3. [file 12864_2022_9062_MOESM3_ESM.pdf]

Additional File 3: Figure S2.
Hexbin plot of Skin Reflectance and natural log transformed Skin Autofluorescence

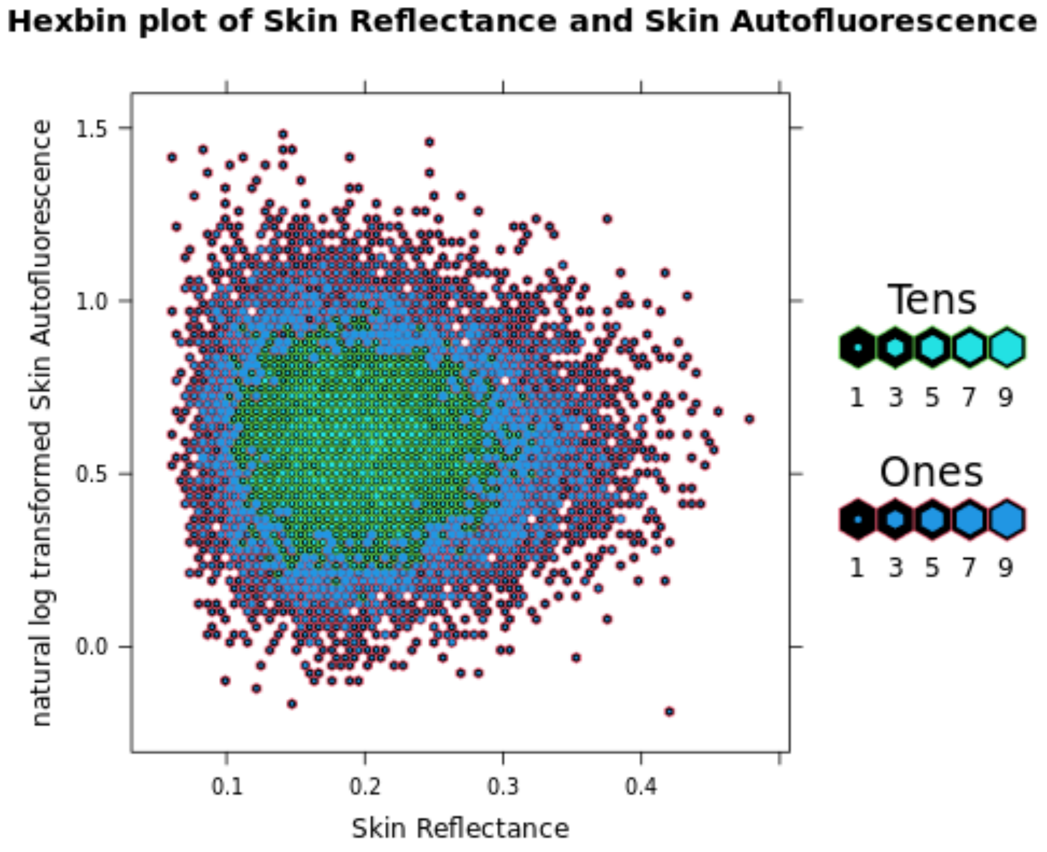

The color of each hexagon corresponds to the mean number of participants in that hexagon.
