# Supplementary material for: Genome-wide association study identifies novel loci associated with skin autofluorescence in individuals without diabetes
Source: BMC Genomics. 2022 Dec 19;23:840. doi: 10.1186/s12864-022-09062-x (PMC9764523; doi:10.1186/s12864-022-09062-x)
Supplement: Supplementary file 4 — Additional file 4. [file 12864_2022_9062_MOESM4_ESM.pdf]

Additional File 4: Table S1.

IMPUTE2 info scores by cohort.

SNP	GSA	CytoSNP
rs12931267	1	0.96
rs3764257	0.99	0.99
rs2846707	0.99	0.999
rs2470893	1	0.83
rs576201050	0.92	0.91
rs1495741	1	0.99

An info score of 1 indicates a genotyped SNP. SNPs with info score >0.8 were included in GWAS.
