# Supplementary material for: Genome-wide association study identifies novel loci associated with skin autofluorescence in individuals without diabetes
Source: BMC Genomics. 2022 Dec 19;23:840. doi: 10.1186/s12864-022-09062-x (PMC9764523; doi:10.1186/s12864-022-09062-x)
Supplement: Supplementary file 6 — Additional file 6. [file 12864_2022_9062_MOESM6_ESM.pdf]

Additional File 6: Table S3.

Linear regression models with rs2470893 and interaction coffee intake/eGFR model 4 GSA.

	Model 4 + rs12931267			Model 4 + rs12931267 + interaction coffee			Model 4 + rs12931267 + interaction eGFR		
**Covariate**	**Beta**	**SE**	**P-value**	**Beta**	**SE**	**P-value**	**Beta**	**SE**	**P-value**
**Age**	1.0×10^{-02}	6.1×10^{-04}	$<2 \times 10^{-16}$	1.0×10^{-02}	6.1×10^{-04}	$<2 \times 10^{-16}$	1.0×10^{-02}	6.1×10^{-04}	$<2 \times 10^{-16}$
**Age squared**	-7.6×10^{-06}	6.8×10^{-06}	2.7×10^{-01}	-7.5×10^{-06}	6.8×10^{-06}	2.7×10^{-01}	-7.6×10^{-06}	6.8×10^{-06}	2.7×10^{-01}
**Male sex**	1.0×10^{-02}	2.6×10^{-03}	1.3×10^{-04}	1.0×10^{-02}	2.6×10^{-03}	1.3×10^{-04}	1.0×10^{-02}	2.6×10^{-03}	1.3×10^{-04}
**Smoking status non-smoker**	Ref	-	-	Ref	-	-	Ref	-	-
**previous smoker**	2.9×10^{-02}	3.4×10^{-03}	$<2 \times 10^{-16}$	2.9×10^{-02}	3.4×10^{-03}	$<2 \times 10^{-16}$	2.9×10^{-02}	3.4×10^{-03}	$<2 \times 10^{-16}$
**current smoker**	6.5×10^{-02}	3.3×10^{-03}	$<2 \times 10^{-16}$	6.5×10^{-02}	3.3×10^{-03}	$<2 \times 10^{-16}$	6.5×10^{-02}	3.3×10^{-03}	$<2 \times 10^{-16}$
**BMI**	1.8×10^{-03}	3.2×10^{-04}	1.3×10^{-08}	1.8×10^{-03}	3.2×10^{-04}	1.4×10^{-08}	1.8×10^{-03}	3.2×10^{-04}	1.3×10^{-08}
**eGFR**	-5.4×10^{-04}	1.1×10^{-04}	4.5×10^{-07}	-5.4×10^{-04}	1.1×10^{-04}	4.3×10^{-07}	-5.2×10^{-04}	1.9×10^{-04}	6.2×10^{-03}
**rs1495741 copies G-allele**	-3.8×10^{-02}	2.8×10^{-03}	$<2 \times 10^{-16}$	-3.8×10^{-02}	2.8×10^{-03}	$<2 \times 10^{-16}$	-3.8×10^{-02}	2.8×10^{-03}	$<2 \times 10^{-16}$
**rs1495741 heterozygosity**	-2.4×10^{-02}	3.4×10^{-03}	7.1×10^{-12}	-2.4×10^{-02}	3.4×10^{-03}	7.5×10^{-12}	-2.4×10^{-02}	3.4×10^{-03}	7.1×10^{-12}
**Inclusion method**									
**Family doctor**	Ref	-	-	Ref	-	-	Ref	-	-
**Included family members**	-6.3×10^{-03}	3.0×10^{-03}	3.5×10^{-02}	-6.3×10^{-03}	3.0×10^{-03}	3.5×10^{-02}	-6.3×10^{-03}	3.0×10^{-03}	3.5×10^{-02}
**Self-administrated**	-1.2×10^{-02}	3.9×10^{-03}	1.6×10^{-03}	-1.2×10^{-02}	3.9×10^{-03}	1.7×10^{-03}	-1.2×10^{-02}	3.9×10^{-03}	1.6×10^{-03}
**HbA1c**	1.5×10^{-03}	4.3×10^{-04}	5.0×10^{-04}	1.5×10^{-03}	4.3×10^{-04}	5.0×10^{-04}	1.5×10^{-03}	4.3×10^{-04}	5.0×10^{-04}
**Coffee drinking status**	1.3×10^{-02}	4.4×10^{-03}	3.8×10^{-03}	1.3×10^{-02}	4.4×10^{-03}	3.8×10^{-03}	1.3×10^{-02}	4.4×10^{-03}	3.8×10^{-03}
**cups per day**	1.5×10^{-02}	7.1×10^{-04}	$<2 \times 10^{-16}$	1.6×10^{-02}	1.2×10^{-03}	$<2 \times 10^{-16}$	1.5×10^{-02}	7.1×10^{-04}	$<2 \times 10^{-16}$
**Reflectance**	1.9×10^{-01}	1.9×10^{-02}	$<2 \times 10^{-16}$	1.9×10^{-01}	1.9×10^{-02}	$<2 \times 10^{-16}$	1.9×10^{-01}	1.9×10^{-02}	$<2 \times 10^{-16}$
**rs2470893 C**	-7.5×10^{-03}	1.8×10^{-03}	4.2×10^{-05}	-5.2×10^{-03}	3.2×10^{-03}	1.0×10^{-01}	-6.4×10^{-03}	1.2×10^{-02}	5.9×10^{-01}
**interaction rs2470893 and cups per day**	-	-	-	-6.7×10^{-04}	7.9×10^{-04}	4.0×10^{-01}	-	-	-
**interaction rs2470893 and eGFR**	-	-	-	-	-	-	-1.0×10^{-05}	1.2×10^{-04}	9.3×10^{-01}
**Adjusted R-squared**	0.49			0.49			0.49		
