# Supplementary material for: Genome-wide association study identifies novel loci associated with skin autofluorescence in individuals without diabetes
Source: BMC Genomics. 2022 Dec 19;23:840. doi: 10.1186/s12864-022-09062-x (PMC9764523; doi:10.1186/s12864-022-09062-x)
Supplement: Supplementary file 7 — Additional file 7. [file 12864_2022_9062_MOESM7_ESM.pdf]

Additional File 7: Table S4.

Linear regression models with rs576201050 and interaction rs1495741 model 4 GSA.

	Model 4 + rs576201050			Model 4 + rs576201050 + interaction		
**Covariate**	**Beta**	**SE**	**P-value**	**Beta**	**SE**	**P-value**
**Age**	1.0×10^{-02}	6.1×10^{-04}	$<2 \times 10^{-16}$	1.0×10^{-02}	6.1×10^{-04}	$<2 \times 10^{-16}$
**Age squared**	-6.9×10^{-06}	6.8×10^{-06}	3.1×10^{-01}	-7.0×10^{-06}	6.8×10^{-06}	3.1×10^{-01}
**Male sex**	9.7×10^{-03}	2.6×10^{-03}	2.3×10^{-04}	9.8×10^{-03}	2.6×10^{-03}	2.0×10^{-04}
**Smoking status**						
**non-smoker**	Ref	-	-	Ref	-	-
**previous smoker**	2.9×10^{-02}	3.4×10^{-03}	$<2 \times 10^{-16}$	2.9×10^{-02}	3.4×10^{-03}	$<2 \times 10^{-16}$
**current smoker**	6.4×10^{-02}	3.3×10^{-03}	$<2 \times 10^{-16}$	6.4×10^{-02}	3.3×10^{-03}	$<2 \times 10^{-16}$
**BMI**	1.8×10^{-03}	3.2×10^{-04}	1.4×10^{-08}	1.8×10^{-03}	3.2×10^{-04}	1.6×10^{-08}
**eGFR**	-5.3×10^{-04}	1.1×10^{-04}	7.7×10^{-07}	-5.2×10^{-04}	1.1×10^{-04}	7.9×10^{-07}
**rs1495741 copies G-allele**	-3.8×10^{-02}	2.8×10^{-03}	$<2 \times 10^{-16}$	-3.8×10^{-02}	2.8×10^{-03}	$<2 \times 10^{-16}$
**rs1495741 heterozygosity**	-2.4×10^{-02}	3.4×10^{-03}	8.3×10^{-12}	-2.4×10^{-02}	3.4×10^{-03}	2.5×10^{-12}
**Inclusion method**						
**Family doctor**	Ref	-	-	Ref	-	-
**Included family members**	-6.4×10^{-03}	3.0×10^{-03}	3.0×10^{-02}	-6.4×10^{-03}	3.0×10^{-03}	3.2×10^{-02}
**Self-administrated**	-1.2×10^{-02}	3.9×10^{-03}	1.7×10^{-03}	-1.2×10^{-02}	3.9×10^{-03}	1.7×10^{-03}
**HbA1c**	1.5×10^{-03}	4.3×10^{-04}	4.8×10^{-04}	1.5×10^{-03}	4.3×10^{-04}	4.3×10^{-04}
**Coffee drinking status**	1.2×10^{-02}	4.4×10^{-03}	6.7×10^{-03}	1.2×10^{-02}	4.4×10^{-03}	6.8×10^{-03}
**cups per day**	1.6×10^{-02}	7.1×10^{-04}	$<2 \times 10^{-16}$	1.6×10^{-02}	7.1×10^{-04}	$<2 \times 10^{-16}$
**Reflectance**	1.9×10^{-01}	1.9×10^{-02}	$<2 \times 10^{-16}$	1.9×10^{-01}	1.9×10^{-02}	$<2 \times 10^{-16}$
**rs576201050 A**	-4.3×10^{-02}	8.5×10^{-03}	3.5×10^{-07}	-5.4×10^{-02}	9.7×10^{-03}	2.2×10^{-08}
**interaction rs12931267 and rs1495741**	-	-	-	4.7×10^{-02}	2.0×10^{-02}	1.9×10^{-02}
**Adjusted R-squared**	0.49			0.49		
