# Supplementary material for: Genome-wide association study identifies novel loci associated with skin autofluorescence in individuals without diabetes
Source: BMC Genomics. 2022 Dec 19;23:840. doi: 10.1186/s12864-022-09062-x (PMC9764523; doi:10.1186/s12864-022-09062-x)
Supplement: Supplementary file 8 — Additional file 8. [file 12864_2022_9062_MOESM8_ESM.docx]

**Additional File 8: Table S5.**

**Genome-wide associations for skin reflectance**

| **SNP** | **CHR:BP** | **EA** | **AA** | **EAF _GSA_** | **B _META_** | **SE _META_** | ***P* _META_** | **Nearby gene** | ***P* _SAF_** |
| --- | --- | --- | --- | --- | --- | --- | --- | --- | --- |
| rs535930 | 1:110724488 | A | G | 0.52 | 0.003 | 0.0005 | 4.3 × 10^−9^ | *SLC6A17* | 0.07 |
| rs35805265 | 2:119631981 | T | C | 0.36 | -0.003 | 0.0005 | 4.1 × 10^−9^ | *MARCO* | 0.06 |
| rs6792049 | 3:156353161 | T | C | 0.56 | -0.003 | 0.0005 | 3.9 × 10^−10^ | *TIPARP* | 0.22 |
| rs35407 | 5:33946571 | A | G | 0.02 | -0.023 | 0.0019 | 1.4 × 10^−34^ | *SLC45A2* | 0.001 ^*^ |
| rs17110447 | 5:149192846 | A | G | 0.70 | 0.004 | 0.0006 | 4.3 × 10^−15^ | *PPARGC1B* | 8.4 × 10^−5 *^ |
| rs12203592 | 6:396321 | T | C | 0.06 | 0.015 | 0.0010 | 2.8 × 10^−47^ | *IRF4* | 1.5 × 10^−5 *^ |
| rs2049865 | 8:116588546 | A | C | 0.58 | -0.003 | 0.0005 | 9.1 × 10^−10^ | *TRPS1* | 0.47 |
| rs12350739 | 9:16885017 | A | G | 0.57 | 0.006 | 0.0005 | 2.5 × 10^−27^ | *BNC2* | 0.004 |
| rs35563099 | 10:119572403 | T | C | 0.14 | -0.008 | 0.0007 | 1.0 × 10^−29^ | *EMX2* | 0.001 ^*^ |
| rs1126809 | 11:89017961 | A | G | 0.24 | 0.009 | 0.0006 | 1.2 × 10^−45^ | *TYR* | 0.0003 ^*^ |
| rs1290177 | 13:113536132 | T | C | 0.44 | -0.003 | 0.0005 | 2.1 × 10^−9^ | *ATP11A* | 0.04 |
| rs121918166 | 15:28230247 | T | C | 0.008 | 0.035 | 0.0032 | 7.4 × 10^−28^ | *HERC2/OCA2* | 0.04 ^*±^ |
| rs35096708 | 16:89887249 | A | G | 0.26 | 0.015 | 0.0006 | 4.5 × 10^−151^ | *MC1R* | 2.8 × 10^−20 *^ |
| rs6059655 | 20:32665748 | A | G | 0.09 | 0.013 | 0.0009 | 1.2 × 10^−49^ | *RALY/ASIP* | 4.5 × 10^−5 *^ |

The top-associated SNP is reported at each locus, along with the genomic coordinates (CHR:BP; GRCh37.p13), the effect allele (EA), the effect allele frequency (EAF), the beta (B) and standard error (SE) from meta-analysis model adjusted for covariates, the meta-analysis *P* value (*P*_META_), the association *P* value in the GSA set (*P*_GSA_) and the *P* value in the CytoSNP set (*P*_CYTO_). *P_SAF_* is the *P* value of the SNP association with SAF in meta-analysis of model 1. P <0.003 (after Bonferroni correction) is considered significantly associated with SAF. * Indicates same direction of effect. ^±^ Indicates association was only tested in the GSA cohort as a result of MAF <0.005 in the CytoSNP cohort. An additional bivariate trait GWAS of SAF and SR using GEMMA software did not reveal any additional significant SNPs that are associated with both traits.
