# Supplementary material for: Genome-wide association study identifies novel loci associated with skin autofluorescence in individuals without diabetes
Source: BMC Genomics. 2022 Dec 19;23:840. doi: 10.1186/s12864-022-09062-x (PMC9764523; doi:10.1186/s12864-022-09062-x)
Supplement: Supplementary file 9 — Additional file 9. [file 12864_2022_9062_MOESM9_ESM.pdf]

Additional File 9: Table S6.
Independent association signals resulting from stepwise conditional analysis for Skin Reflectance in the *MC1R* region

SNP	CHR:BP	EA	AA	EAF $_{GSA}$	B $_{META}$	SE $_{META}$	P $_{META}$	P $_{SAF}$
rs35096708	16:89887249	A	G	0.26	0.015	0.0006	4.5×10^{-151}	2.8×10^{-20} *
rs8049897	16:90024202	A	G	0.13	0.013	0.0008	5.0×10^{-63}	5.8×10^{-15}
rs1805008	16:89986144	T	C	0.10	0.015	0.0010	6.3×10^{-50}	1.4×10^{-6} *
rs150909008	16:90004693	A	G	0.13	0.012	0.0008	1.7×10^{-52}	0.004 *
rs117990130	16:89787340	T	C	0.18	-0.012	0.0010	8.4×10^{-30}	0.16
rs190295257	16:89906879	C	G	0.01	0.025	0.0027	5.3×10^{-20}	2.6×10^{-5} *
rs55637757	16:89535888	T	C	0.12	0.0079	0.0009	5.8×10^{-20}	0.0007 *
rs1805006	16:89985918	A	C	0.01	0.023	0.0026	2.8×10^{-19}	0.003 *
rs117204628	16:90032455	T	C	0.01	0.018	0.0022	2.5×10^{-15}	0.003 *
rs1805007	16:89986117	T	C	0.07	0.013	0.0017	1.2×10^{-14}	2.3×10^{-20} *
rs62052172	16:89986091	A	G	0.004	0.029	0.0038	4.1×10^{-14}	0.11 *
rs885479	16:89986154	A	G	0.05	0.0086	0.0013	3.7×10^{-11}	0.04
rs11547464	16:89950230	C	G	0.12	0.0057	0.0010	2.3×10^{-9}	0.007 *
rs1805007	16:89986546	C	G	0.008	0.033	0.0055	2.1×10^{-9} ±	0.006 *±

The SNPs are reported by rsID and genomic coordinates (CHR:BP; GRCh37.p13), the effect allele (EA), alternate allele (AA), the effect allele frequency (EAF) is reported, the beta (B_{META}) and standard error (SE_{META}) and *P* value (P_{META}) are from stepwise conditional analysis on all SNPs in rows above. P_{SAF} is the *P* value of the SNP association with SAF in meta-analysis of model 1. * Indicates same direction of effect. ± Indicates association was only tested in the GSA cohort as a result of MAF <0.005 in the CytoSNP cohort.
