# Supplementary material for: Genome-wide association study identifies novel loci associated with skin autofluorescence in individuals without diabetes
Source: BMC Genomics. 2022 Dec 19;23:840. doi: 10.1186/s12864-022-09062-x (PMC9764523; doi:10.1186/s12864-022-09062-x)

Additional File 10: Figure S3.

Manhattan and QQ-plot for skin reflectance.
Covariates in the model: Age, sex, source of inclusion, BMI, smoking group, eGFR and month of measurement.

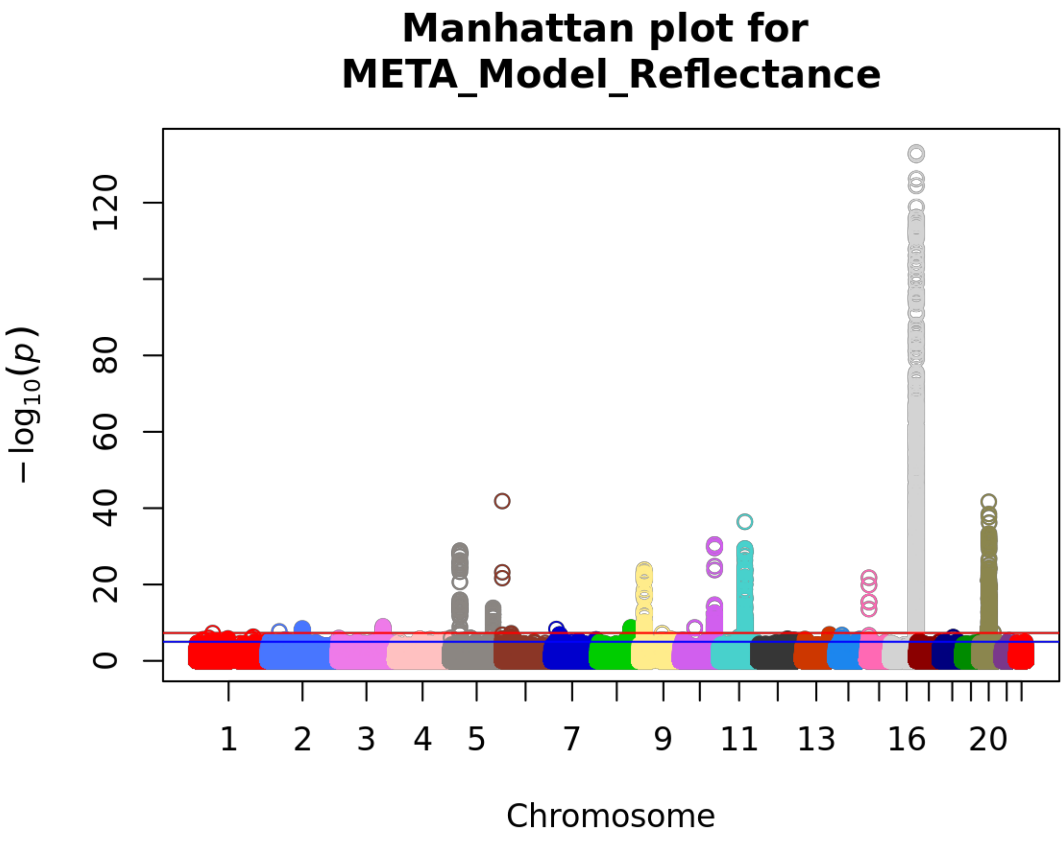

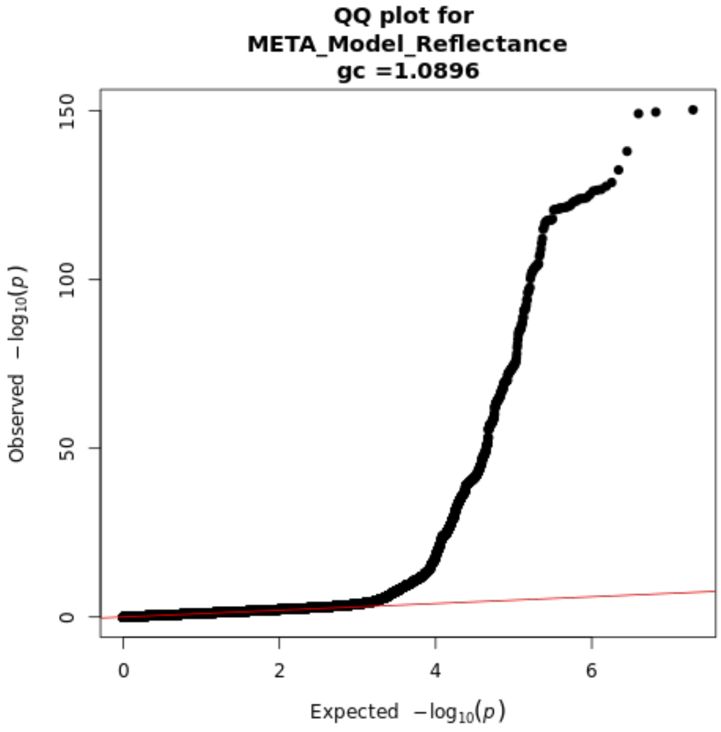

Supplement: Supplementary file 10 — Additional file 10. [file 12864_2022_9062_MOESM10_ESM.pdf]
