# Supplementary material for: Genome-wide association study identifies novel loci associated with skin autofluorescence in individuals without diabetes
Source: BMC Genomics. 2022 Dec 19;23:840. doi: 10.1186/s12864-022-09062-x (PMC9764523; doi:10.1186/s12864-022-09062-x)
Supplement: Supplementary file 11 — Additional file 11. [file 12864_2022_9062_MOESM11_ESM.docx]

**Additional File 11: Table S7.**

**Association of SNPs associated with SAF tested for association with Skin Reflectance**

| **SNP** | **CHR:BP** | **EA** | **AA** | **B _META_** | **SE _META_** | ***P* _META_** | **Nearby gene** |
| --- | --- | --- | --- | --- | --- | --- | --- |
| rs12931267 | 16:89818732 | C | G | -0.0236 | 0.0010 | 3.0 × 10^−127^ * | *FANCA* |
| rs2846707 | 11:102576358 | T | C | 0.0002 | 0.0005 | 0.66 | *MMP27* |
| rs2470893 | 15:75019449 | T | C | 0.0001 | 0.0005 | 0.91 * | *CYP1A1* |
| rs576201050 | 8:18288053 | A | G | -0.0007 | 0.0025 | 0.79 * | *NAT2* |
| rs1495741 | 8:18272881 | A | G | -0.0015 | 0.0006 | 0.01 | *NAT2* |
| rs3764257 | 16:89800887 | C | G | -0.0091 | 0.0006 | 1.0 × 10^−57^ * | *ZNF276* |

The top-associated SNP is reported at each locus, along with the genomic coordinates (CHR:BP; GRCh37.p13), the effect allele (EA), , the beta (B) and standard error (SE) from meta-analysis of Skin Reflectance model adjusted for covariates, the meta-analysis *P* value (*P*_META_), * Indicates same direction of effect. An additional bivariate trait GWAS of SAF and SR using GEMMA software did not reveal any additional significant SNPs that are associated with both traits.
