# Supplementary material for: Genome-wide association study identifies novel loci associated with skin autofluorescence in individuals without diabetes
Source: BMC Genomics. 2022 Dec 19;23:840. doi: 10.1186/s12864-022-09062-x (PMC9764523; doi:10.1186/s12864-022-09062-x)
Supplement: Supplementary file 12 — Additional file 12. [file 12864_2022_9062_MOESM12_ESM.pdf]

**Additional File 12: Table S8.**
**Association of known SNPs associated with coffee consumption and plasma caffeine metabolites with skin autofluorescence**[1,2]

| SNP | CHR:BP | EA/AA | EAF GSA | B META | SE META | P META | Nearby gene |
|---|---|---|---|---|---|---|---|
| rs574367 | 1:177873210 | T/G | 0.20 | 0.0011 | 0.0018 | 0.53 | *SEC16B* |
| **rs10865548** | **2:631606** | **G/A** | **0.84** | **0.0057** | **0.0019** | **0.0032 *** | ***TMEM18*** |
| rs1260326 | 2:27730940 | C/T | 0.65 | 0.000039 | 0.0015 | 0.98 | *GCKR* |
| rs117692895 | 7:172776925 | C/G | x | x | x | x | *AHR* |
| **rs4410790** | **7:17284577** | **C/T** | **0.64** | **0.0064** | **0.0015** | **$1.5 \times 10^{-5}$ *** | ***AHR*** |
| rs4719497 | 7:17334899 | T/C | 0.88 | 0.0020 | 0.0021 | 0.35 * | *AHR* |
| rs12699844 | 7:17443199 | C/T | 0.50 | 0.0023 | 0.0014 | 0.098 * | *KCCAT333* |
| **rs73073176** | **7:17562952** | **C/T** | **0.89** | **0.0055** | **0.0022** | **0.014 *** | ***LOC101927630*** |
| rs34060476 | 7:73037956 | G/A | 0.14 | 0.0010 | 0.002 | 0.63 * | *MLXIPL* |
| **rs1057868** | **7:75615006** | **T/C** | **0.30** | **0.0035** | **0.0015** | **0.024 *** | ***POR*** |
| rs597045 | 11:56272114 | A/T | 0.70 | -0.0016 | 0.0015 | 0.29 | *OR8U8* |
| rs1956218 | 14:33075243 | G/A | 0.56 | 0.0009 | 0.0014 | 0.55 | *AKAP6* |
| **rs2472297** | **15:75027880** | **T/C** | **0.27** | **0.0107** | **0.002** | **$5.6 \times 10^{-8}$ *±** | ***CYP1A1/2*** |
| rs66723169 | 18:57808978 | A/C | 0.25 | 0.0021 | 0.0016 | 0.20 * | *MC4R* |
| **rs2330783** | **22:24747031** | **G/T** | **0.98** | **0.0151** | **0.0046** | **0.0011 *** | ***SPECC1L-ADORA2A*** |
| rs1481012 | 4:89039082 | A/G | 0.88 | 0.0016 | 0.0021 | 0.46 | *ABCG2* |
| rs62391270 | 6:14161434 | T/C | 0.82 | -0.0012 | 0.0019 | 0.52 | *CD83* |
| rs56113850 | 19:41353107 | T/C | 0.44 | -0.0017 | 0.0018 | 0.33± | *CYP2A6* |

EA: effect allele, AA: alternate allele * Indicates same direction of effect. ± Indicates association was only tested in the GSA cohort as a result of INFO < 0.8 in the CytoSNP cohort. X means results not available.

The SNPs that are marked in a box are additional SNPs that were previously associated with plasma caffeine metabolites from Cornelis et al.,

[1] Zhong VW, Kuang A, Danning RD, Kraft P, Van Dam RM, Chasman DI, et al. A genome-wide association study of bitter and sweet beverage consumption. Hum Mol Genet 2019;28:2449. https://doi.org/10.1093/HMG/DDZ061.
[2] Cornelis MC, Kacprowski T, Menni C, Gustafsson S, Pivin E, Adamski J, et al. Genome-wide association study of caffeine metabolites provides new insights to caffeine metabolism and dietary caffeine-consumption behavior. Hum Mol Genet 2016;25:5472–82. https://doi.org/10.1093/HMG/DDW334.
