# Supplementary material for: Genome-wide association study identifies novel loci associated with skin autofluorescence in individuals without diabetes
Source: BMC Genomics. 2022 Dec 19;23:840. doi: 10.1186/s12864-022-09062-x (PMC9764523; doi:10.1186/s12864-022-09062-x)
Supplement: Supplementary file 13 — Additional file 13. [file 12864_2022_9062_MOESM13_ESM.pdf]

Additional File 13: Table S9.

Single-Tissue eQTLs for rs12931267, chromosome 16, effect allele G

	Gene Symbol	P-Value	NES	Tissue
**DBNDD1**				
	DBNDD1	2.9E-29	0.86	Artery - Tibial
	DBNDD1	3.2E-27	0.80	Nerve - Tibial
	DBNDD1	7.8E-27	0.73	Lung
	DBNDD1	2.3E-26	0.60	Thyroid
	DBNDD1	5.0E-26	1.2	Colon - Transverse
	DBNDD1	1.0E-24	1.0	Heart - Atrial Appendage
	DBNDD1	1.2E-21	0.60	Skin - Sun Exposed (Lower leg)
	DBNDD1	2.3E-21	0.63	Heart - Left Ventricle
	DBNDD1	6.4E-21	1.0	Colon - Sigmoid
	DBNDD1	1.5E-20	0.58	Esophagus - Muscularis
	DBNDD1	1.9E-20	1.0	Cells - Cultured fibroblasts
	DBNDD1	2.1E-19	0.76	Adipose - Visceral (Omentum)
	DBNDD1	3.0E-18	0.69	Skin - Not Sun Exposed (Suprapubic)
	DBNDD1	1.8E-16	0.64	Pancreas
	DBNDD1	2.8E-16	0.55	Stomach
	DBNDD1	1.8E-14	0.72	Adrenal Gland
	DBNDD1	5.3E-14	0.74	Prostate
	DBNDD1	6.3E-14	0.60	Adipose - Subcutaneous
	DBNDD1	2.4E-10	0.56	Esophagus - Gastroesophageal Junction
	DBNDD1	1.1E-09	0.87	Artery - Coronary
	DBNDD1	1.9E-08	0.56	Pituitary
	DBNDD1	6.5E-08	0.93	Small Intestine - Terminal Ileum
	DBNDD1	1.2E-07	0.42	Artery - Aorta
	DBNDD1	2.1E-06	0.25	Breast - Mammary Tissue
	DBNDD1	3.9E-05	-0.20	Muscle - Skeletal
	DBNDD1	7.4E-05	0.46	Spleen
**GAS8**				
	GAS8	1.7E-20	1.4	Brain - Cerebellum
	GAS8	1.1E-13	1.3	Brain - Cerebellar Hemisphere
	GAS8	1.5E-12	-0.56	Muscle - Skeletal
	GAS8	1.9E-10	0.65	Adrenal Gland

	GAS8	7.7E-10	0.46	Skin - Sun Exposed (Lower leg)
	GAS8	1.0E-09	0.84	Brain - Cortex
	GAS8	3.0E-08	0.89	Brain - Frontal Cortex (BA9)
	GAS8	6.8E-08	0.54	Pituitary
	GAS8	1.7E-06	0.35	Skin - Not Sun Exposed (Suprapubic)
	GAS8	1.9E-06	0.81	Brain - Putamen (basal ganglia)
	GAS8	6.7E-06	0.58	Brain - Caudate (basal ganglia)
	GAS8	2.8E-05	0.62	Brain - Anterior cingulate cortex (BA24)
**GAS8-AS1**				
	GAS8-AS1	1.3E-18	1.5	Brain - Cerebellum
	GAS8-AS1	2.2E-10	1.1	Brain - Cerebellar Hemisphere
	GAS8-AS1	1.6E-08	0.98	Brain - Cortex
	GAS8-AS1	1.9E-08	1.2	Brain - Frontal Cortex (BA9)
	GAS8-AS1	2.0E-05	0.46	Adipose - Subcutaneous
	GAS8-AS1	3.7E-05	0.73	Brain - Nucleus accumbens (basal ganglia)
	GAS8-AS1	6.0E-05	0.79	Brain - Anterior cingulate cortex (BA24)
**FANCA**				
	FANCA	2.4E-11	-0.23	Cells - Cultured fibroblasts
	FANCA	1.4E-09	-0.24	Esophagus - Mucosa
	FANCA	1.9E-08	-0.40	Testis
	FANCA	4.0E-07	-0.22	Skin - Sun Exposed (Lower leg)
	FANCA	7.1E-07	-0.32	Spleen
	FANCA	1.1E-06	-0.54	Adrenal Gland
	FANCA	3.9E-06	-0.23	Skin - Not Sun Exposed (Suprapubic)
	FANCA	7.5E-06	-0.38	Breast - Mammary Tissue
	FANCA	1.7E-05	-0.30	Thyroid
	FANCA	3.1E-05	-0.47	Brain - Cerebellum
	FANCA	3.4E-05	-0.39	Cells - EBV-transformed lymphocytes
	FANCA	5.5E-05	-0.27	Lung
	FANCA	6.1E-05	-0.44	Brain - Cortex
	FANCA	1.2E-04	-0.35	Artery - Aorta
**CHMP1A**				
	CHMP1A	2.7E-10	-0.45	Lung
	CHMP1A	2.0E-08	-0.42	Nerve - Tibial
	CHMP1A	7.2E-08	-0.46	Thyroid

	CHMP1A	1.3E-06	-0.13	Whole Blood
	CHMP1A	7.0E-06	-0.26	Artery - Tibial
	CHMP1A	8.1E-06	-0.37	Artery - Aorta
	CHMP1A	8.7E-06	-0.30	Adipose - Subcutaneous
	CHMP1A	1.5E-05	-0.32	Esophagus - Muscularis
	CHMP1A	2.6E-05	-0.37	Testis
	CHMP1A	6.0E-05	-0.34	Spleen
	CHMP1A	9.1E-05	-0.18	Skin - Sun Exposed (Lower leg)
**SPIRE2**				
	SPIRE2	2.1E-08	-0.48	Muscle - Skeletal
	SPIRE2	7.4E-08	-0.39	Esophagus - Mucosa
	SPIRE2	1.6E-06	-0.40	Esophagus - Muscularis
	SPIRE2	2.3E-06	-0.30	Thyroid
	SPIRE2	7.0E-06	-0.49	Adrenal Gland
	SPIRE2	9.6E-06	-0.39	Pituitary
	SPIRE2	1.2E-05	-0.47	Testis
	SPIRE2	1.4E-05	-0.29	Breast - Mammary Tissue
	SPIRE2	2.7E-05	-0.31	Skin - Sun Exposed (Lower leg)
	SPIRE2	3.6E-05	-0.47	Esophagus - Gastroesophageal Junction
	SPIRE2	3.9E-05	-0.45	Artery - Aorta
	SPIRE2	6.2E-05	-0.35	Artery - Tibial
**CDK10**				
	CDK10	4.2E-08	-0.40	Testis
	CDK10	4.1E-07	-0.27	Skin - Not Sun Exposed (Suprapubic)
	CDK10	1.4E-05	-0.26	Nerve - Tibial
	CDK10	5.4E-05	-0.19	Thyroid
	CDK10	7.2E-05	-0.21	Lung
**SPATA33**				
	SPATA33	5.8E-08	-0.34	Whole Blood
**VPS9D1**				
	VPS9D1	2.6E-06	-0.19	Cells - Cultured fibroblasts
	VPS9D1	1.8E-04	-0.20	Skin - Sun Exposed (Lower leg)
**URAHP**				
	URAHP	3.0E-06	0.67	Brain - Cerebellum
	URAHP	5.6E-06	0.67	Brain - Cerebellar Hemisphere

	URAHP	1.4E-05	-0.32	Esophagus - Mucosa
	URAHP	4.5E-05	0.30	Heart - Left Ventricle
**AC137932.6**				
	AC137932.6	1.9E-05	-0.36	Heart - Left Ventricle
**LINC02166**				
	LINC02166	2.4E-05	0.40	Testis
**RP11-104N10.2**				
	RP11-104N10.2	4.9E-05	-0.31	Thyroid
**CPNE7**				
	CPNE7	5.5E-05	-0.42	Pituitary
	CPNE7	1.0E-04	-0.34	Cells - Cultured fibroblasts
**RP11-368I7.6**				
	RP11-368I7.6	6.3E-05	-0.55	Pituitary
**DPEP1**				
	DPEP1	1.6E-04	0.40	Nerve - Tibial

NES: Normalized effect size; a positive value indicates increased expression of the gene for every copy of the minor allele and a negative NES indicates decreased expression of the gene for every copy of the minor allele.

We utilized data from the Genotype-Tissue Expression (GTEx) project (Release v8, dbGaP Accession phs000424.v8.p2 available at: http://www.gtexportal.org)[3].
