# Supplementary material for: Genome-wide association study identifies novel loci associated with skin autofluorescence in individuals without diabetes
Source: BMC Genomics. 2022 Dec 19;23:840. doi: 10.1186/s12864-022-09062-x (PMC9764523; doi:10.1186/s12864-022-09062-x)
Supplement: Supplementary file 14 — Additional file 14. [file 12864_2022_9062_MOESM14_ESM.pdf]

Additional File 14: Table S10.

Single-Tissue eQTLs for rs2846707, chromosome 11, effect allele C

	**Gene Symbol**	**P-Value**	**NES**	**Tissue**
**MMP27**				
	MMP27	7.7E-41	-0.35	Skin - Sun Exposed (Lower leg)
	MMP27	8.7E-31	-0.39	Skin - Not Sun Exposed (Suprapubic)
	MMP27	2.3E-29	-0.33	Adipose - Subcutaneous
	MMP27	1.0E-17	-0.37	Muscle - Skeletal
	MMP27	3.3E-10	-0.31	Breast - Mammary Tissue
	MMP27	9.6E-10	-0.28	Esophagus - Muscularis
	MMP27	1.7E-09	0.22	Whole Blood
	MMP27	8.2E-09	-0.57	Liver
	MMP27	1.6E-08	-0.19	Cells - Cultured fibroblasts
	MMP27	1.6E-08	-0.26	Adipose - Visceral (Omentum)
	MMP27	9.7E-08	-0.25	Nerve - Tibial
	MMP27	2.2E-07	-0.48	Minor Salivary Gland
	MMP27	2.8E-07	-0.24	Colon - Transverse
	MMP27	7.6E-07	-0.27	Esophagus - Gastroesophageal Junction
	MMP27	2.6E-06	-0.24	Esophagus - Mucosa
**RP11-817J15.3**				
	RP11-817J15.3	1.4E-12	-0.67	Liver
	RP11-817J15.3	2.7E-07	-0.47	Small Intestine - Terminal Ileum
**TMEM123**				
	TMEM123	7.7E-05	-0.097	Cells - Cultured fibroblasts

NES: Normalized effect size; a positive value indicates increased expression of the gene for every copy of the minor allele and a negative NES indicates decreased expression of the gene for every copy of the minor allele.
