# Supplementary material for: Genome-wide association study identifies novel loci associated with skin autofluorescence in individuals without diabetes
Source: BMC Genomics. 2022 Dec 19;23:840. doi: 10.1186/s12864-022-09062-x (PMC9764523; doi:10.1186/s12864-022-09062-x)
Supplement: Supplementary file 15 — Additional file 15. [file 12864_2022_9062_MOESM15_ESM.pdf]

Additional File 15: Table S11.

Single-Tissue eQTLs for rs2470893, chromosome 15, effect allele T

	Gene Symbol	P-Value	NES	Tissue
**RPP25**				
	RPP25	1.6E-14	-0.31	Cells - Cultured fibroblasts
	RPP25	4.6E-14	-0.31	Artery - Tibial
	RPP25	2.1E-12	-0.27	Nerve - Tibial
	RPP25	7.4E-11	-0.36	Artery - Aorta
	RPP25	5.6E-10	-0.18	Lung
	RPP25	7.3E-10	-0.21	Adipose - Subcutaneous
	RPP25	1.2E-06	-0.15	Thyroid
	RPP25	1.9E-05	-0.19	Heart - Atrial Appendage
	RPP25	7.9E-05	-0.17	Breast - Mammary Tissue
	RPP25	1.0E-04	-0.14	Esophagus - Muscularis
**SCAMP2**				
	SCAMP2	5.3E-12	0.16	Esophagus - Mucosa
	SCAMP2	8.0E-11	0.15	Skin - Sun Exposed (Lower leg)
	SCAMP2	6.7E-10	0.10	Whole Blood
	SCAMP2	3.0E-09	0.15	Skin - Not Sun Exposed (Suprapubic)
	SCAMP2	7.7E-08	0.33	Testis
**MPI**				
	MPI	2.6E-09	0.19	Cells - Cultured fibroblasts
	MPI	8.8E-08	0.17	Esophagus - Mucosa
	MPI	2.5E-07	0.17	Nerve - Tibial
	MPI	7.9E-06	0.28	Brain - Putamen (basal ganglia)
	MPI	2.9E-05	0.28	Brain - Caudate (basal ganglia)
	MPI	3.4E-05	0.18	Breast - Mammary Tissue
	MPI	1.0E-04	0.19	Stomach
	MPI	1.4E-04	0.082	Whole Blood
	MPI	2.1E-04	0.13	Esophagus - Muscularis
**SCAMP5**				
	SCAMP5	7.0E-08	-0.25	Thyroid

	SCAMP5	1.9E-06	-0.22	Nerve - Tibial
	SCAMP5	3.5E-06	-0.21	Artery - Tibial
	SCAMP5	1.1E-04	-0.22	Cells - Cultured fibroblasts
	SCAMP5	1.3E-04	-0.25	Artery - Aorta
**RP11-60L3.2**				
	RP11-60L3.2	4.2E-07	0.26	Testis
**ULK3**				
	ULK3	2.1E-06	-0.22	Pancreas
	ULK3	1.1E-05	-0.096	Skin - Not Sun Exposed (Suprapubic)
	ULK3	1.4E-05	-0.11	Cells - Cultured fibroblasts
**MAN2C1**				
	MAN2C1	2.6E-06	-0.15	Whole Blood
	MAN2C1	6.6E-06	-0.18	Artery - Tibial
	MAN2C1	6.7E-05	-0.18	Lung
	MAN2C1	7.6E-05	-0.16	Skin - Not Sun Exposed (Suprapubic)
	MAN2C1	1.4E-04	-0.20	Artery - Aorta
	MAN2C1	1.9E-04	-0.17	Cells - Cultured fibroblasts
	MAN2C1	2.6E-04	-0.15	Thyroid
**SNUPN**				
	SNUPN	6.0E-06	-0.16	Cells - Cultured fibroblasts
	SNUPN	8.6E-05	-0.12	Nerve - Tibial
	SNUPN	1.0E-04	-0.18	Testis
	SNUPN	1.0E-04	-0.10	Muscle - Skeletal
	SNUPN	1.4E-04	-0.18	Heart - Atrial Appendage
	SNUPN	3.2E-04	-0.11	Thyroid
**GOLGA6C**				
	GOLGA6C	1.5E-05	0.24	Testis
**SEMA7A**				
	SEMA7A	5.9E-05	-0.20	Testis
**ISLR2**				
	ISLR2	5.9E-05	0.23	Esophagus - Mucosa
	ISLR2	2.4E-04	0.18	Adipose - Subcutaneous
**FAM219B**				
	FAM219B	7.2E-05	0.10	Adipose - Subcutaneous

**PPCDC**				
	PPCDC	1.1E-04	-0.11	Artery - Tibial
**CYP1A1**				
	CYP1A1	2.1E-04	-0.22	Muscle - Skeletal

NES: Normalized effect size; a positive value indicates increased expression of the gene for every copy of the minor allele and a negative NES indicates decreased expression of the gene for every copy of the minor allele.
