# Supplementary material for: Genome-wide association study identifies novel loci associated with skin autofluorescence in individuals without diabetes
Source: BMC Genomics. 2022 Dec 19;23:840. doi: 10.1186/s12864-022-09062-x (PMC9764523; doi:10.1186/s12864-022-09062-x)
Supplement: Supplementary file 16 — Additional file 16. [file 12864_2022_9062_MOESM16_ESM.pdf]

Additional File 16: Table S12.

Single-Tissue eQTLs for rs3764257, chromosome 16, effect allele G

	Gene Symbol	P-Value	NES	Tissue
**CDK10**				
	CDK10	1E-26	-0.29	Muscle - Skeletal
	CDK10	8.1E-25	-0.36	Artery - Tibial
	CDK10	4.4E-24	-0.32	Adipose - Subcutaneous
	CDK10	2.4E-23	-0.33	Nerve - Tibial
	CDK10	1E-22	-0.22	Whole Blood
	CDK10	1.1E-20	-0.33	Esophagus - Muscularis
	CDK10	2.5E-20	-0.25	Thyroid
	CDK10	6.2E-18	-0.32	Adipose - Visceral (Omentum)
	CDK10	5.6E-17	-0.23	Skin - Sun Exposed (Lower leg)
	CDK10	3.6E-14	-0.23	Lung
	CDK10	9.5E-14	-0.35	Artery - Aorta
	CDK10	1.8E-13	-0.33	Breast - Mammary Tissue
	CDK10	7.1E-13	-0.23	Skin - Not Sun Exposed (Suprapubic)
	CDK10	8E-13	-0.21	Esophagus - Mucosa
	CDK10	1.6E-12	-0.22	Heart - Left Ventricle
	CDK10	3.1E-12	-0.31	Colon - Sigmoid
	CDK10	4.3E-12	-0.33	Esophagus - Gastroesophageal Junction
	CDK10	2.2E-11	-0.35	Adrenal Gland
	CDK10	3.9E-11	-0.26	Heart - Atrial Appendage
	CDK10	2.8E-09	-0.24	Stomach
	CDK10	5.2E-09	-0.29	Cells - Cultured fibroblasts
	CDK10	7.6E-09	-0.65	Cells - EBV-transformed lymphocytes
	CDK10	8E-09	-0.19	Colon - Transverse
	CDK10	1.3E-08	-0.26	Prostate
	CDK10	2.3E-08	-0.33	Small Intestine - Terminal Ileum
	CDK10	2.4E-08	-0.37	Minor Salivary Gland
	CDK10	2.6E-08	-0.37	Spleen
	CDK10	2.9E-08	-0.38	Vagina
	CDK10	3E-08	-0.24	Testis
	CDK10	5.1E-08	-0.37	Brain - Cerebellum
	CDK10	1E-07	-0.24	Pancreas

	CDK10	2.2E-07	-0.32	Ovary
	CDK10	2.6E-07	-0.22	Pituitary
	CDK10	4.3E-07	-0.34	Uterus
	CDK10	0.000015	-0.23	Brain - Frontal Cortex (BA9)
	CDK10	0.000032	-0.31	Brain - Cerebellar Hemisphere
**CENPBD1**				
	CENPBD1	0.0000097	0.32	Brain - Cerebellar Hemisphere
	CENPBD1	0.000036	0.18	Adipose - Visceral (Omentum)
	CENPBD1	0.00020	0.17	Nerve - Tibial
**CHMP1A**				
	CHMP1A	9.1E-08	0.21	Cells - Cultured fibroblasts
	CHMP1A	3.1E-07	0.16	Esophagus - Mucosa
	CHMP1A	8.7E-07	0.13	Skin - Sun Exposed (Lower leg)
**CPNE7**				
	CPNE7	0.00011	-0.23	Nerve - Tibial
**DEF8**				
	DEF8	0.000053	-0.12	Skin - Not Sun Exposed (Suprapubic)
**DPEP1**				
	DPEP1	0.000041	-0.43	Adrenal Gland
**FANCA**				
	FANCA	1.6E-12	-0.19	Whole Blood
	FANCA	2E-09	-0.23	Lung
	FANCA	1.4E-07	-0.22	Thyroid
	FANCA	0.000038	0.086	Cells - Cultured fibroblasts
	FANCA	0.000087	-0.29	Pituitary
	FANCA	0.00010	0.16	Testis
**LINC02166**				
	LINC02166	0.000048	0.49	Spleen
	LINC02166	0.00014	0.29	Cells - Cultured fibroblasts
**MC1R**				
	MC1R	1.1E-09	-0.26	Cells - Cultured fibroblasts
	MC1R	1.4E-07	0.16	Skin - Sun Exposed (Lower leg)
	MC1R	1.5E-07	0.22	Whole Blood
	MC1R	0.0000012	0.23	Esophagus - Mucosa
	MC1R	0.00025	0.16	Skin - Not Sun Exposed (Suprapubic)

**SPATA2L**				
	SPATA2L	5.6E-07	0.18	Esophagus - Mucosa
**SPIRE2**				
	SPIRE2	5.6E-13	0.30	Skin - Sun Exposed (Lower leg)
	SPIRE2	1.3E-09	0.26	Esophagus - Mucosa
	SPIRE2	0.0000090	-0.16	Lung
	SPIRE2	0.000021	0.17	Brain - Hippocampus
	SPIRE2	0.000043	0.23	Brain - Anterior cingulate cortex (BA24)
	SPIRE2	0.000054	0.21	Brain - Nucleus accumbens (basal ganglia)
**TCF25**				
	TCF25	1.7E-12	0.25	Esophagus - Mucosa
	TCF25	4.2E-09	0.14	Whole Blood
**TUBB3**				
	TUBB3	0.0000010	0.23	Esophagus - Mucosa
	TUBB3	0.00010	-0.17	Breast - Mammary Tissue
**VPS9D1**				
	VPS9D1	1.8E-40	0.36	Esophagus - Mucosa
	VPS9D1	2.7E-37	0.36	Thyroid
	VPS9D1	1.1E-33	0.34	Nerve - Tibial
	VPS9D1	1.8E-29	0.35	Adipose - Subcutaneous
	VPS9D1	2.3E-27	0.32	Skin - Sun Exposed (Lower leg)
	VPS9D1	1.1E-26	0.35	Lung
	VPS9D1	8.7E-20	0.36	Testis
	VPS9D1	1.3E-19	0.30	Skin - Not Sun Exposed (Suprapubic)
	VPS9D1	2.5E-18	0.32	Heart - Atrial Appendage
	VPS9D1	7.3E-17	0.29	Adipose - Visceral (Omentum)
	VPS9D1	3.5E-15	0.46	Spleen
	VPS9D1	5.3E-15	0.32	Breast - Mammary Tissue
	VPS9D1	7.3E-14	0.33	Prostate
	VPS9D1	1.3E-13	0.26	Heart - Left Ventricle
	VPS9D1	1.6E-13	0.19	Esophagus - Muscularis
	VPS9D1	3.6E-11	0.20	Artery - Tibial
	VPS9D1	1.3E-10	0.30	Pituitary
	VPS9D1	7.3E-10	0.20	Colon - Transverse
	VPS9D1	2.1E-09	0.28	Brain - Nucleus accumbens (basal ganglia)

	VPS9D1	2.3E-09	0.15	Muscle - Skeletal
	VPS9D1	3.1E-09	0.30	Pancreas
	VPS9D1	7.9E-09	0.25	Artery - Aorta
	VPS9D1	8.5E-09	0.23	Brain - Cortex
	VPS9D1	2.7E-08	0.10	Whole Blood
	VPS9D1	3.5E-08	0.24	Brain - Hypothalamus
	VPS9D1	1.2E-07	0.23	Brain - Hippocampus
	VPS9D1	1.5E-07	0.32	Artery - Coronary
	VPS9D1	1.6E-07	0.17	Esophagus - Gastroesophageal Junction
	VPS9D1	1.9E-07	0.26	Brain - Anterior cingulate cortex (BA24)
	VPS9D1	9.6E-07	0.33	Small Intestine - Terminal Ileum
	VPS9D1	0.0000014	0.23	Stomach
	VPS9D1	0.0000016	0.25	Brain - Caudate (basal ganglia)
	VPS9D1	0.0000043	0.22	Brain - Frontal Cortex (BA9)
	VPS9D1	0.000011	0.25	Brain - Putamen (basal ganglia)
	VPS9D1	0.000020	0.33	Minor Salivary Gland
	VPS9D1	0.000022	0.26	Ovary
	VPS9D1	0.000026	0.099	Cells - Cultured fibroblasts
	VPS9D1	0.000052	0.16	Colon - Sigmoid
	VPS9D1	0.00011	0.16	Brain - Cerebellum
**VPS9D1-AS1**				
	VPS9D1-AS1	5.1E-33	0.38	Whole Blood
	VPS9D1-AS1	2.3E-26	0.49	Thyroid
	VPS9D1-AS1	6.3E-25	0.50	Adipose - Subcutaneous
	VPS9D1-AS1	2.5E-16	0.36	Lung
	VPS9D1-AS1	1.2E-14	0.34	Nerve - Tibial
	VPS9D1-AS1	2.2E-14	0.45	Colon - Sigmoid
	VPS9D1-AS1	3.9E-13	0.47	Heart - Atrial Appendage
	VPS9D1-AS1	1.7E-12	0.42	Artery - Aorta
	VPS9D1-AS1	3.2E-12	0.35	Adipose - Visceral (Omentum)
	VPS9D1-AS1	1.5E-11	0.34	Esophagus - Muscularis
	VPS9D1-AS1	3E-10	0.29	Artery - Tibial
	VPS9D1-AS1	1.7E-09	0.39	Brain - Caudate (basal ganglia)
	VPS9D1-AS1	2.2E-09	0.57	Spleen
	VPS9D1-AS1	5.1E-09	0.39	Pituitary
	VPS9D1-AS1	6.6E-09	0.28	Muscle - Skeletal

	VPS9D1-AS1	6.6E-09	0.36	Heart - Left Ventricle
	VPS9D1-AS1	9.1E-08	0.40	Brain - Cortex
	VPS9D1-AS1	1.2E-07	0.35	Brain - Nucleus accumbens (basal ganglia)
	VPS9D1-AS1	3.4E-07	0.34	Testis
	VPS9D1-AS1	8.3E-07	0.17	Esophagus - Mucosa
	VPS9D1-AS1	0.0000026	0.31	Esophagus - Gastroesophageal Junction
	VPS9D1-AS1	0.0000078	0.15	Colon - Transverse
	VPS9D1-AS1	0.0000078	0.36	Minor Salivary Gland
	VPS9D1-AS1	0.000018	0.34	Brain - Putamen (basal ganglia)
	VPS9D1-AS1	0.000023	0.38	Brain - Hypothalamus
	VPS9D1-AS1	0.000024	0.32	Small Intestine - Terminal Ileum
	VPS9D1-AS1	0.000052	0.30	Brain - Cerebellar Hemisphere
	VPS9D1-AS1	0.000059	0.33	Brain - Frontal Cortex (BA9)
	VPS9D1-AS1	0.000089	0.23	Brain - Cerebellum
**ZNF276**				
	ZNF276	2E-12	-0.38	Brain - Cerebellum
	ZNF276	3E-11	-0.23	Nerve - Tibial
	ZNF276	5E-10	-0.18	Muscle - Skeletal
	ZNF276	7.1E-09	-0.37	Brain - Cerebellar Hemisphere
	ZNF276	0.0000016	-0.27	Brain - Putamen (basal ganglia)
	ZNF276	0.0000065	-0.24	Brain - Nucleus accumbens (basal ganglia)
	ZNF276	0.000021	-0.18	Breast - Mammary Tissue
	ZNF276	0.000023	-0.20	Adrenal Gland
	ZNF276	0.000088	-0.11	Thyroid

NES: Normalized effect size; a positive value indicates increased expression of the gene for every copy of the minor allele and a negative NES indicates decreased expression of the gene for every copy of the minor allele.

We utilized data from the Genotype-Tissue Expression (GTEx) project (Release v8, dbGaP Accession phs000424.v8.p2 available at: http://www.gtexportal.org)[3].
